# The Dynamic Changes of the Plasma Membrane Proteins and the Protective Roles of Nitric Oxide in Rice Subjected to Heavy Metal Cadmium Stress

**DOI:** 10.3389/fpls.2016.00190

**Published:** 2016-02-26

**Authors:** Liming Yang, Jianhui Ji, Karen R. Harris-Shultz, Hui Wang, Hongliang Wang, Elsayed F. Abd-Allah, Yuming Luo, Xiangyang Hu

**Affiliations:** ^1^Jiangsu Key Laboratory for Eco-Agriculture Biotechnology around Hongze Lake, Jiangsu Collaborative Innovation Center of Regional Modern Agriculture and Environment Protection, Huaiyin Normal UniversityHuaian, China; ^2^Department of Plant Pathology, University of GeorgiaTifton, GA, USA; ^3^Crop Protection and Management Research Unit, United States Department of Agriculture, Agricultural Research ServiceTifton, GA, USA; ^4^Crop Genetics and Breeding Research Unit, United States Department of Agriculture, Agricultural Research ServiceTifton, GA, USA; ^5^Department of Plant Production, Faculty of Food and Agricultural Sciences, King Saud UniversityRiyadh, Saudi Arabia; ^6^Shanghai Key Laboratory of Bio-Energy Crops, School of Life Sciences, Shanghai UniversityShanghai, China

**Keywords:** rice, cadmium pollution, nitric oxide, reactive oxygen species, lipid hydrolysis, quantitative proteomics

## Abstract

The heavy metal cadmium is a common environmental contaminant in soils and has adverse effects on crop growth and development. The signaling processes in plants that initiate cellular responses to environmental stress have been shown to be located in the plasma membrane (PM). A better understanding of the PM proteome in response to environmental stress might provide new insights for improving stress-tolerant crops. Nitric oxide (NO) is reported to be involved in the plant response to cadmium (Cd) stress. To further investigate how NO modulates protein changes in the plasma membrane during Cd stress, a quantitative proteomics approach based on isobaric tags for relative and absolute quantification (iTRAQ) was used to identify differentially regulated proteins from the rice plasma membrane after Cd or Cd and NO treatment. Sixty-six differentially expressed proteins were identified, of which, many function as transporters, ATPases, kinases, metabolic enzymes, phosphatases, and phospholipases. Among these, the abundance of phospholipase D (PLD) was altered substantially after the treatment of Cd or Cd and NO. Transient expression of the *PLD* fused with green fluorescent peptide (GFP) in rice protoplasts showed that the Cd and NO treatment promoted the accumulation of PLD in the plasma membrane. Addition of NO also enhanced Cd-induced PLD activity and the accumulation of phosphatidic acid (PA) produced through PLD activity. Meanwhile, NO elevated the activities of antioxidant enzymes and caused the accumulation of glutathione, both which function to reduce Cd-induced H_2_O_2_ accumulation. Taken together, we suggest that NO signaling is associated with the accumulation of antioxidant enzymes, glutathione and PA which increases cadmium tolerance in rice via the antioxidant defense system.

## Introduction

The heavy metal cadmium is a common environmental contaminant with a long biological half-life in the soil and has adverse effects on crop growth and development (Das et al., 1997; Tuan Anh and Popova, 2013; Choppala et al., 2014). Cadmium pollution is caused by the application of phosphate fertilizer and certain industrial processes and poses a critical threat to human health due to its over-accumulation in the soil and in crop food (Prasad, 1995; Das et al., 1997; Zhang et al., 2015).

Cadmium is absorbed by plant roots and is quickly transported to the leaves via the xylem. Most plants are very sensitive to trace amounts of cadmium, responding with retardation in growth and development, due to decreased photosynthesis in the leaves. This impairs photosynthetic supply and accelerates apoptosis and necrosis of the leaves. In plants, cadmium can be detoxified by phytochelatins, a class of glutathione-derived peptides containing repeating units of Glu and Cys that function by binding metal ions and transporting them to the vacuole (Howden et al., 1995; Di Toppi and Gabbrielli, 1999). Plant cells also can use antioxidant enzymes to degrade over-produced reactive oxygen species (ROS) to diminish damage from cadmium exposure (Cobbett et al., 1998; Singh and Tewari, 2003; Balestrasse et al., 2006). In addition, nitric oxide (NO), an important signaling molecule, participates in the cadmium stress response in plants and protects against cadmium-induced damage in *Helianthus* (Laspina et al., 2005) and aluminum toxicity in rice (Yang et al., 2013). Furthermore, NO is required for cadmium-induced programmed cell death in *Arabidopsis* (Balestrasse et al., 2006; De Michele et al., 2009).

The plasma membrane of a plant cell is a highly organized system that mediates the exchange of information and materials between the cell interior and the extracellular environment. The enzymes or molecules located in the plasma membrane play important roles in transferring stress signals in plants. Phospholipases, including phospholipase D (PLD), phospholipase C (PLC), and phospholipase A (PLA), play an important role in lipid hydrolysis of the plasma membrane and in the mediation of the stress response in plants (Wang et al., 2002; Wang, 2006). For example, heavy metal stress from copper ions can trigger PLD activity in wheat roots (Wang et al., 2002; Navari-Izzo et al., 2006). In *Arabidopsis*, phosphatidic acid (PA) produced by PLD interacts with NADPH oxidase in the plasma membrane to generate H_2_O_2_ in response to abscisic acid (ABA; Wang, 2006; Zhang et al., 2009). However, the cross-talk between NO, PLD activity, and the antioxidant system during the stress response to heavy metals remains uncharacterized.

The plasma membrane is the first site of extracellular biotic or abiotic sensing, so an understanding of proteome dynamics may facilitate the development of new strategies for stress resistance in crops (Sussman, 1994; Santoni et al., 1998; Alexandersson et al., 2004). As the primary environmental barrier, the plasma membrane of a plant cell controls many biological processes such as ion transport, endocytosis, cell differentiation and proliferation, and signal transduction. However, functional protein localization is complicated because some membrane proteins are tightly associated with the dual lipid core, whereas others are loosely and reversibly associated (Sussman and Harper, 1989; Sussman, 1994). Technological advances in the extraction of purified plant plasma membrane proteins have made it possible to profile the proteome of the entire plant membrane (Santoni et al., 2000; Alexandersson et al., 2004). Proteome profiling may elucidate which systemic protective responses are initiated in plant plasma membranes exposed to environmental stress.

To investigate the accumulation patterns of proteins localized in the plasma membranes of rice seedlings exposed to cadmium, we conducted an iTRAQ-based quantitative proteomic analysis for proteins in the plasma membrane. We found that cadmium treatment induced a rapid increase in a set of transporter proteins and ATPases, as well as a PLD protein. Subsequent experiments showed that cadmium induced rapid generation of NO and PA (produced assumedly through PLD activity). The exogenous addition of either NO or PA with Cd mitigated cadmium toxicity in rice seedlings and augmented synthesis of glutathione that functions to reduce oxidative damage. Based on these results, we propose a model that depicts how NO and PLD improves Cd tolerance in rice seedlings.

## Materials and methods

### Growth of rice seedlings and cadmium treatment

Seeds of the rice variety (*Oryza sativa* ssp. Japonica cv Zhonghua11) were surface-sterilized, washed, and germinated on wet filter paper. Seeds were then grown in 1/4 Hoagland's nutrient solution (pH 5.5) at 23°C and 16 h light/8 h dark conditions. When the third leaves of the seedlings emerged, CdCl_2_ was added to the culture solution to a final concentration of 10 μM. To examine the impact of NO, rice seedlings were pretreated with a 30 μM solution of S-nitroso-N-acetylpenicillamine (SNAP), a spontaneous NO donor, for 2 h prior to cadmium exposure. The SNAP solution was refreshed every 3 days, and the pH of solution was maintained at 5.5. Aliquots of the seedlings were harvested for subsequent assays at the indicated times. For the inhibitor treatment, different inhibitors including 2-(4-carboxyphenyl)-4,4,5,5- tetra methylimidazoline-1-oxyl-3-oxide (cPTIO), and 1-butanol (1-Bu) were added into the rice culture solution for 2 h prior to cadmium treatment, respectively.

### Extraction and purification of plasma membranes from rice seedlings

The rice plasma membrane was enriched by aqueous two phase partitioning as previously described (Nohzadeh Malakshah et al., 2007). In brief, about 10 g of rice seedlings were ground to a powder in liquid nitrogen and homogenized in 50 ml of ice-cold 50 mM 3-(N-morpholino) propanesulfonic acid (MOPS)/KOH buffer (pH 7.5) containing 330 mM sucrose, 5 mM EDTA, 5 mM DTT, 5 mM ascorbate, 0.5 mM phenyl-methyl-sulfonyl fluoride (PMSF), 0.2% BSA, 0.2% casein, and 0.6% polyvinylpyrrolidone (PVP) at 4°C. The homogenate was centrifuged at 2000 × g for 10 min at 4°C, and the supernatant was filtered through a 260-μm filter. The filtrate was centrifuged again at 12,000 × g for 10 min at 4°C, and the resulting supernatant was centrifuged at 50,000 × g for 60 min at 4°C to precipitate the microsomal pellets. To enrich for plasma membranes, the microsomal pellets were re-suspended in 10 ml of resuspension buffer (330 mM sucrose, 5 mM potassium phosphate [pH 7.8], 2 mM potassium chloride, 1 mM DTT, and 0.1 mM EDTA) and were mixed with a phase mixture containing 6.3% (w/w) Dextran T500 (Sigma-Aldrich, St. Louis, MO, US), 6.3% (w/w) PEG3350 (Sigma-Aldrich), 330 mM sucrose, 5 mM phosphate buffer [pH 7.8], 1 mM KCl, 0.5 mM EDTA, and 1 mM DTT to yield a 36-g phase system. After mixing, phase separation was conducted at 4°C. The upper (PEG) phase containing a second nascent partitioning was further purified into two sub-phases. The upper phase of this partition was diluted with wash buffer (250 mM sucrose, 10 mM MOPS) and then centrifuged at 100,000 × g for 60 min. The resulting plasma membrane pellets were dissolved into 1 ml of sample buffer containing 330 mM sucrose and 50 mM 2-(N-morpholino) ethanesulfonic acid (MES)/KOH (pH 6.0). The purity of the isolated plasma membranes was evaluated by measuring the activities of several marker enzymes for different membrane fractions.

### Protein digestion, iTRAQ labeling, and protein quantification

The proteins of the plasma membrane were prepared for iTRAQ labeling as previously described (Kong et al., 2014), and then was dissolved in 1% SDS, 100 mM triethylammonium bicarbonate, pH 8.5, followed by reduction, alkylation, trypsin digestion, and labeling using 8-plex iTRAQ reagent kits, according to the manufacturer's instructions (AB Sciex, Framingham, MA). Labeled samples were lyophilized, and the peptide mixture was dissolved in a strong cation exchange (SCX) solvent A (25% v/v acetonitrile, 10 mM ammonium formate, pH 2.8). The resulting peptides were fractionated on an Agilent HPLC system 1100 with a polysulfethyl A column (2.1 × 100 mm, 5 μm, 300 A, PolyLC, Columbia, MD). Peptides were eluted at a flow rate of 200 μL/min with a linear gradient of 0-20% of solvent B consisting of 25% v/v acetonitrile, 500 mM ammonium formate for over 50 min, followed by ramping up to 100% solvent B for 5 min and holding for 10 min. The absorbance at 214 nm was monitored, and a total of 12 fractions were collected. Each SCX fraction was lyophilized and dissolved in solvent A (3% acetonitrile v/v, 0.1% formic acid v/v) and then was analyzed with a Q-Exactive Hybrid Quadrupole-Orbitrap mass spectrometer (Thermo Finnigan Scientific, San Jose, CA). Samples were separated on a Hypersile Gold C18 column (100 mm × 2.1 mm, 1.9 μm; Thermo Fisher Scientific, Pittsburgh, PA). Peptides were eluted with a linear gradient of acetonitrile/0.1% formic acid from 3 to 50% for 90 min at a flow rate of 250 nL/min. Peptides were then sprayed into the orifice of the Q-Exactive MS/MS system with a spray voltage of 2.2 kV. Full-scan mass spectra were performed over 200–1800 m/z at high resolution at 60,000. At least the four most intense precursor ions were selected for collision-induced fragmentation in the linear ion trap with 50–2000 m/z and 30–2000 ms at a resolution of 7500. Dynamic exclusion was employed within 40 s to prevent repetitive selection of the peptides.

The raw LC-MS/MS files were analyzed using the software, Proteome Discoverer 1.3 (Thermo Fisher Scientific, Pittsburgh, PA), which was connected to the Mascot Search Engine server, version 2.3 (Matrix Science, Boston, MA). The spectra were searched against the NCBInr protein database (Taxonomy: *O. sativa*, which contains 132,343 sequences). Search parameters included iTRAQ 8-plex quantification, carbamidomethylation of cysteine was set as a fixed modification, and oxidation of methionine was set as a variable modification. Trypsin was specified as the proteolytic enzyme, and one missed cleavage was allowed. Peptide mass tolerance was set at 10 ppm; fragment mass tolerance was set at 0.1 Da. An automatic decoy database search was performed as part of the search. False discovery rates (FDRs) for peptide identification of all searches were <1.0%. The data were pre-filtered to exclude MS/MS spectra containing fewer than three peaks. Mascot results were filtered with the Mascot Percolator package to improve the accuracy and sensitivity of peptide identification. For differential analyses, all proteins identified and quantified with at least four independent peptides with a high degree of confidence (FDR 1%) were selected. The quantification was performed by normalizing the results of all of the measured iTRAQ reported ratios value using the software Proteome Discoverer 1.3 software. Only the significant ratios from the replicates were used to calculate the average ratio for the protein. It should be noted that each p value was generated based on quantitative information derived from at least three independent peptides in each replicate. Cut-offs of 1.2- or 0.6-fold were set to indicate up-regulation or down-regulation of proteins, and a *p* < 0.05 was used to indicate significance.

### Analyses of electrolyte leakage and chlorophyll fluorescence analyses

One gram of treated rice seedling roots were excised and transferred to tubes containing 10 ml of deionized water. The conductivity of the solution was measured after shaking overnight at room temperature. After measurement, samples were autoclaved, and the conductivity of the solution was measured again. The percentage of electrolyte leakage was then calculated, as previously described (Xia et al., 2009). The chlorophyll fluorescence was analyzed with a PAM (pulse-amplitude modulation) Chlorophyll Fluorometer (Heinz-Walz-GmbH, Effeltrich, Germany) as previously described (Yang et al., 2013; Ma et al., 2015).

### Analyses of H_2_O_2_, NO, glutathione, and phytochelatin

H_2_O_2_ levels were measured as described previously (Yang et al., 2013). NO levels were detected using the NO-specific fluorescence probe, 4-amino-5-methylamino-2′,7′-difluorofluorescein diacetate (DAF-FMDA), under epifluorescence microscopy (PCM 2000, excitation 488 nm, emission 515–560 nm, Nikon, Tokyo, Japan) or by using a hemoglobin assay (Hu et al., 2005). The levels of glutathione and phytochelatin were analyzed using a reported method (Gupta and Goldsbrough, 1991).

### Analyses of lipid molecular species and PLD activity

After different treatments, about 1 g of rice seedlings was immediately immersed in 3 mL of isopropanol with 0.01% butylated hydroxytoluene to terminate lipolytic activities. Lipid extraction, ESI-MS/MS analysis, and quantification were done as described previously (Zhang et al., 2009). Five replicates of each treatment were carried out and analyzed. The PLD activity was measured as described (Devaiah et al., 2007).

### Measurement of antioxidant enzyme activity

Leaves (1–2 g) were homogenized in 50 mM sodium phosphate buffer (pH 7.0) containing 1.0 mM ethylenediaminetetraacetic acid (EDTA), 0.5% (v/v) Triton X-100 and 1% (w/v) polyvinylpyrrolidone (PVPP; 100 mg tissue/mL buffer). For the analysis of APX, the extraction buffer also contained 5 mM AsA. The homogenates were centrifuged at 15,000 × g for 20 min at 4°C, and the resulting supernatant was immediately used for the antioxidant enzyme assays. The total activities of ascorbate peroxidase (APX, EC1.11.1.11), superoxide dismutase (SOD, EC1.15.1.1), and glutathione reductase (GR, EC1.6.4.2) in the supernatants were determined as described previously (Zhang et al., 2010; Yang et al., 2015).

### Plasmid construction and transient transformation

A reporter protein construct, named GFP-PLDa, comprised of PLD fused with green fluorescent protein (GFP) was generated by amplifying the full-length PLD alpha (PLDa) cDNA (GenBank: NM_001064550) using the primers of 5′GGCCGAATTCatggcggagcagcagctgatgc3′ and 5′GGCCAAGCTTctacgaggt gatatcgggggtca3′. The resulting PCR fragments were digested with EcoRI and HindIII. The digested fragments were inserted into the binary vector pEGAD to fuse with EGFP at C-terminus. The final construct was confirmed by sequencing. The protoplast preparation and transient transformation were manipulated as described previously (Sheen, 2001; Yoo et al., 2007).

### SDS-PAGE and western blotting

Samples (1 g) were ground to a powder in liquid nitrogen, and were homogenized with 10 mL of extraction buffer containing 125 mM Tris-Cl [pH 7.5], 10% SDS, and 10% mercaptoethanol at 4°C. The mixture was centrifuged at 12,000 × g at 4°C for 10 min, and proteins of the resulting supernatant were separated by SDS-PAGE. Following electrophoresis, separated proteins were transferred to a Hybond-C nitrocellulose membrane (GE Life Sciences, Buckinghamshire, UK) using the Multiphor II semi-dry blotting apparatus, according to the manufacturer's instructions (GE Life Sciences). The transferred proteins were visualized using an ECL detection kit (Roche, Mannheim, Germany), and the antibodies against plant SOD (1:2000), GR (1:1000), and APX (1:2000) were obtained from Agrisera (Agrisera, Vannas, Sweden). To detect PLD proteins in the plasma membrane, the rabbit polyclonal anti-PLDa antibody was prepared by immunizing rabbits with the 14-amino acid N-terminal sequence of the rice PLDa protein (MAHLLMHGTLDATI; GenBank: BAD35530) conjugated to the keyhole limpet hemocyanin.

## Results

### NO reduces cadmium toxicity to leaf photosynthesis and increases root growth

**Figure 1:**
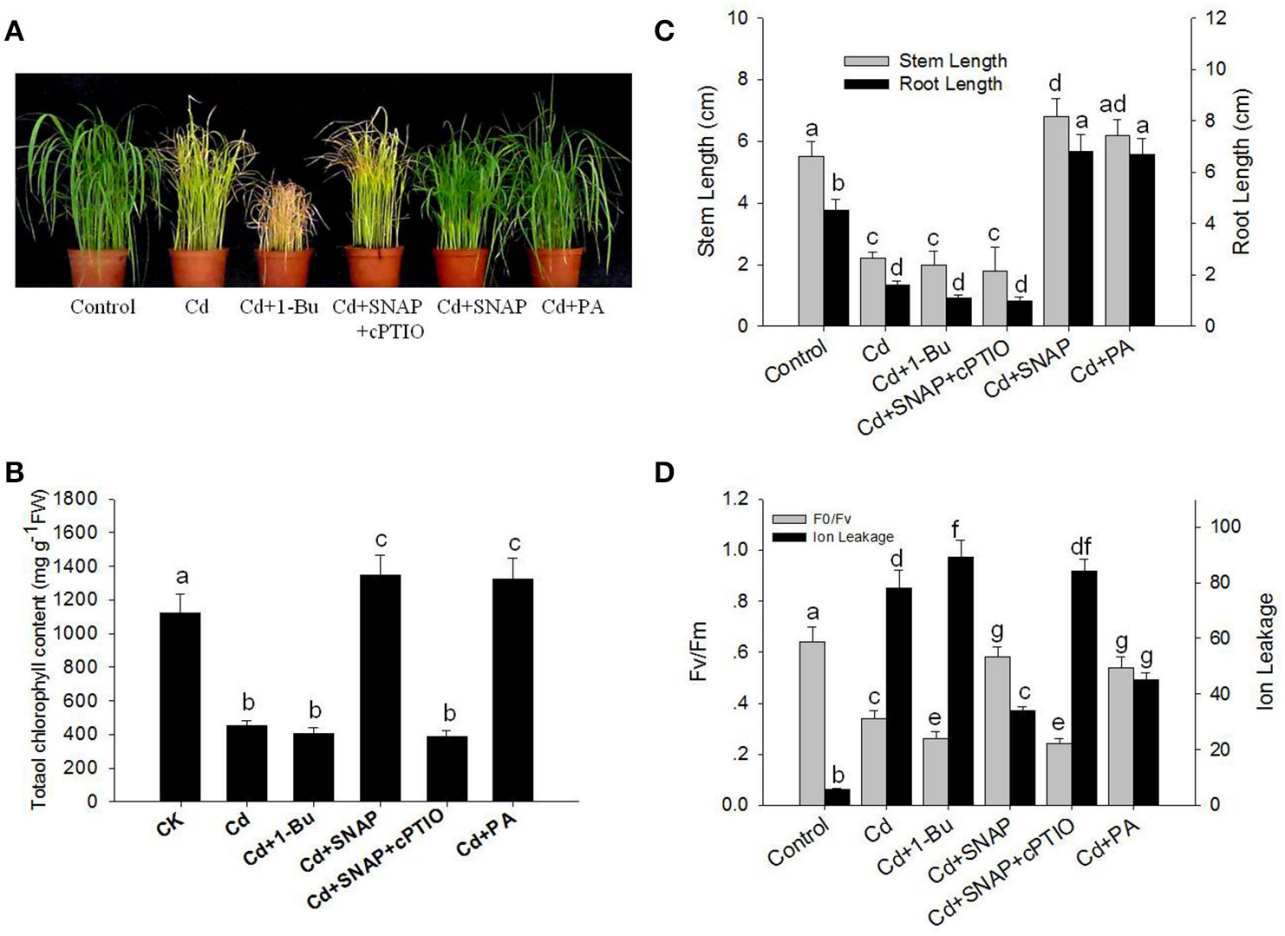
**Effects of cadmium, SNAP, and different chemicals on rice growth and on photosynthetic capability**. These experiments were repeated three times with similar results. **(A)** The rice seedlings at the third leaf stage were treated with 10 μM cadmium (Cd) with or without 30 μM SNAP, 1 μM PA (16:0-18:2), 0.1% 1-Bu, or 30 μM cPTIO for 7 days, and phenotypes from one experiment were noted. **(B)** The effect of cadmium treatment on total chlorophyll content after 7 days of treatment (*n* = 30/experiment). **(C)** The effect of cadmium treatment on stem and root length after 7 days of treatment (*n* = 30/experiment). **(D)** The effects of cadmium treatment on the Fv/Fm ratio and ion leakage in roots after 7 days of treatment. Values reflect means ± SEs of at least three independent experiments (*n* = 30/experiment). Different symbols above the bars indicate significant differences (Tukey's test, *p* < 0.05).

**Figure 2:**
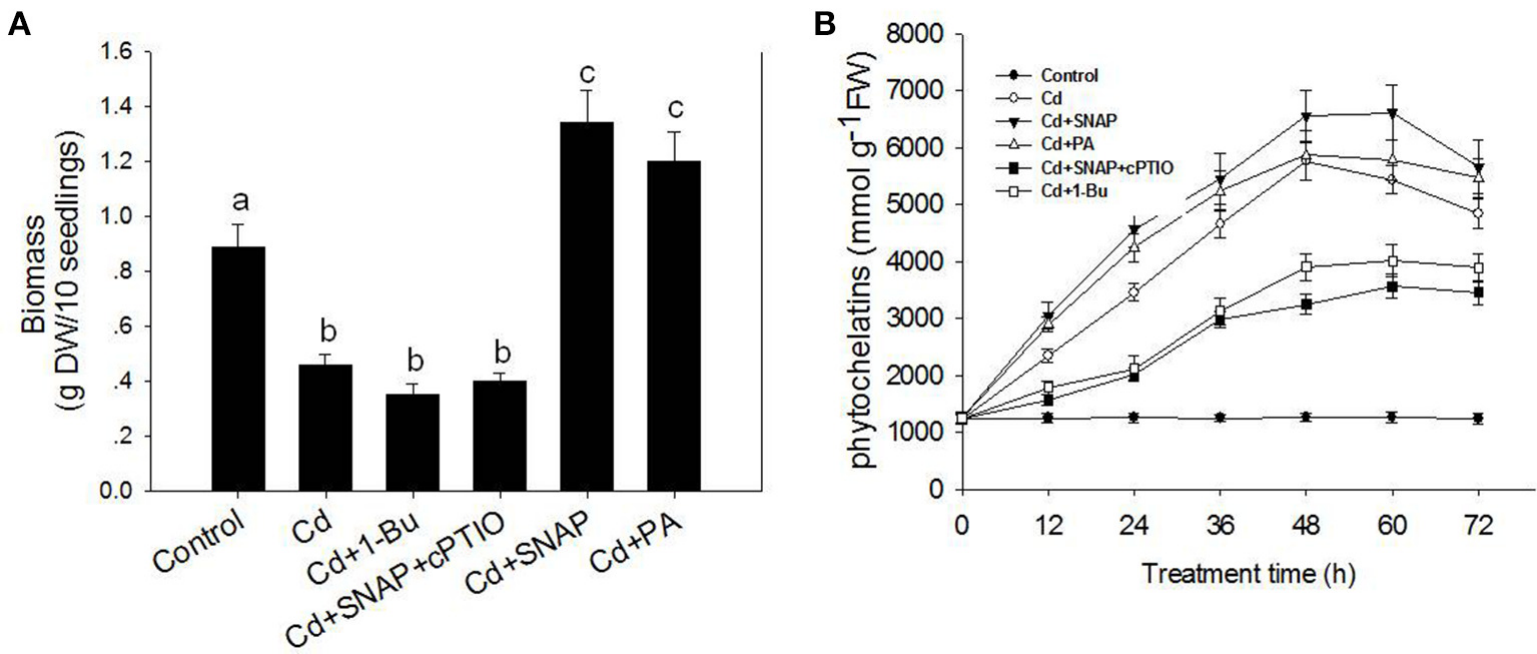
**Effects of cadmium, SNAP, and different chemicals on rice biomass (A) and phytochelatins (B)**. The rice seedlings growth and treatment was the same as in Figure 1, but the biomass and phytochelatin content were measured after 7 day of treatment. Values reflect means ± SEs of three independent experiments (*n* = 30/experiment). Different symbols above the bars indicate significant differences (Tukey's test, *p* < 0.05).

To investigate the effect of cadmium on the growth and development of rice, we first assessed the dose effect of cadmium on 2-week old rice seedlings. As shown in Supplemental Figure 1, increasing the concentration of cadmium decreased shoot and root growth. Seedlings treated with 10 μM of cadmium showed a 50% inhibitory effect on rice growth. Thus the concentration used for cadmium treatment was 10 μM for the subsequent experiments to allow for direct comparisons. As shown in Figure 1A, cadmium treatment for 1 week significantly suppressed rice seedling growth, and caused yellowing of leaves as compared to the control plants. The chlorophyll content in the leaves of rice is an important index to evaluate the photosynthesis capability and plant tolerance to environmental stress. Here we found that Cd treatment for 1 week reduced the chlorophyll content by 59.8% as compared to control plants (Figure 1B). Cd treatment also suppressed the root and stem length by 64.1 and 60.0%, respectively, compared the control seedlings without cadmium treatment (Figure 1C). The ratio of Fv/Fm and ion leakage is a measurement of the photosynthetic capability and the degree of membrane damage, respectively. Cd treatment decreased Fv/Fm from 0.64 to 0.34 and increased the ion leakage by 13.9-fold (Figure 1D). Cd treatment suppressed the biomass of rice by 48.3% compared with the control lines without Cd treatment (Figure 2A). Phytochelatins were increased with cadmium treatment and peaked after 48 h of Cd treatment (Figure 2B). This molecule plays an important role in heavy metal detoxification in plants. These data indicate that Cd treatment for 1 week damaged the rice seedling viability.

NO signaling is reported to reduce the toxic effects of heavy metals on plants. Therefore we investigated the role of NO in the response of rice seedlings to Cd stress. The NO artificial donor SNAP treatment significantly improved seedling tolerance to cadmium by facilitating growth (Figures 1A,C), reducing ion leakage (Figure 1D), increasing leaf chlorophyll synthesis capability (including the Fv/Fm ratio and chlorophyll content; Figures 1B,D), and increasing biomass and phytochelatin content (Figures 2A,B). However, these SNAP effects were abolished when the NO scavenger, 2-(4-carboxyphenyl)-4, 4, 5, 5-tetramethylimidazoline-1-oxyl-3-oxide (cPTIO), was applied (Figures 1, 2). These data suggest that NO plays an essential role in the rice seedling response to Cd treatment.

In addition, PA treatment also significantly improved seedling tolerance to cadmium (Figures 1A,C), increasing total chlorophyll content (Figures 1A,B), and increasing biomass and phytochelatin content (Figures 2A,B). Moreover, the addition of Cd and 1-Bu, an inhibitor of PLD, decreased the total chlorophyll content, stem length, and biomass as compared to control plants (Figures 1A–C, 2A).

**Figure 3:**
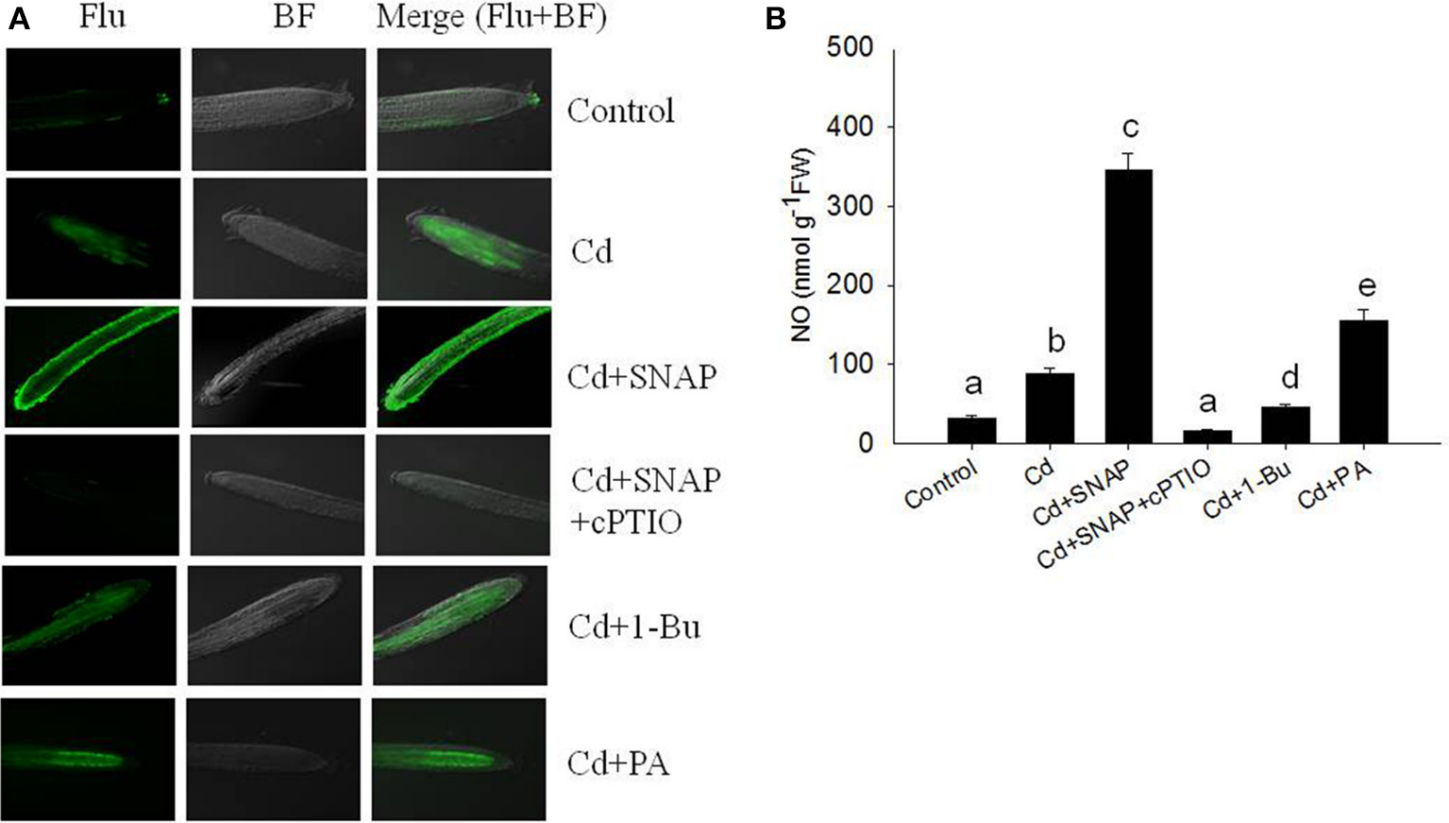
**Effects of cadmium, SNAP, and different chemicals on NO accumulation**. Third-leaf-stage rice seedlings were treated with 10 μM cadmium with or without 30 μM SNAP, 1 μM PA (16:0-18:2), 0.1% 1-Bu, or 30 μM cPTIO. NO fluorescence was detected by DAF-FMDA after 6 h of exposure **(A)**. This experiment was repeated three times with similar results and one replicate is represented. Flu: NO fluorescence; BF: Bright Field. NO content were also measured **(B)**. Values reflect means ± SEs of at least three independent experiments (*n* = 10/experiment). Different symbols above the bars indicate significant differences (Tukey's test, *p* < 0.05).

To further understand the role of NO during the rice response to Cd treatment, we observed the *in-situ* NO accumulation in rice roots subjected to Cd treatment. Using a NO-specific fluorescence probe DAF-FMDA staining, we found that cadmium exposure rapidly increased NO production (Figures 3A,B). NO fluorescence was enhanced with the addition of SNAP and abolished in the presence of cPTIO. By directly measuring the NO content in the roots, we found that Cd treatment increased the NO content as compared to control plants, but this increase could be suppressed by the NO scavenger cPTIO treatment (Figure 3B). Consistent with the NO staining fluorescence results, the addition of Cd and SNAP could further increase NO content. These data suggest that rice seedlings treated with Cd induced NO biosynthesis.

### When plants are treated with cadmium or cadmium and NO, dynamic changes occur in plasma membrane proteins

The plant plasma membrane plays an important role during environmental stress, and most of the previous proteomics studies used total plant proteins to investigate the underlying mechanisms of the plant response to Cd treatment. In this study, we focused on the role of the plasma membrane proteins by isolating plasma membrane proteins and comparing the treatments using iTRAQ (isobaric tags for relative and absolute quantitation) proteomics. To understand the functions of the plasma membrane proteins of rice in stress response, an iTRAQ approach was used to measure the change in plasma membrane proteins isolated from 2-week old seedlings after 12 h or 1 day of 10 μM Cd treatment, or 12 h or 1 day of 10 μM Cd treatment with 30 μM SNAP. The seedlings without Cd or SNAP treatment were used as the control. We isolated and purified the plasma membrane proteins from rice seedlings after Cd treatment using the two-phase partitioning method. The purity of the plasma membrane fraction was evaluated using marker enzymes associated with various subcellular membranes (Natera et al., 2008). Orthovanadate-sensitive ATPase, nitrate-sensitive ATPase, and azide-sensitive ATPase were selected as plasma membrane, vacuolar, and mitochondrial membrane markers, respectively. The ATPase activity of the prepared plasma membrane was primarily sensitive to sodium orthovanadate (Supplemental Table 1). Furthermore, we assessed the purity of the isolated plasma membrane proteins using antibodies against marker proteins corresponding to various subcellular membranes. H^+^-ATPase is a marker protein for the plasma membrane protein fraction, V-ATPase is a tonoplast membrane protein, and molecular chaperone binding protein (BiP) is a protein of the endoplasmic reticulum (ER). Compared with the total protein fraction, the stronger immunoblot signal was detected in the PM fraction using the anti-H^+^-ATPase antibody, suggesting that H^+^-ATPase was strongly enriched in the PM fraction (Supplemental Figure 2). Furthermore, the anti-H^+^-ATPase antibody immunoblotting signal was not detected in other vesicles such as tonoplast, mitochondria, or ER (Supplemental Figure 2). These data suggest that our method is sufficient to obtain high-quality plasma membranes to perform a plasma membrane proteomics study.

**Figure 4:**
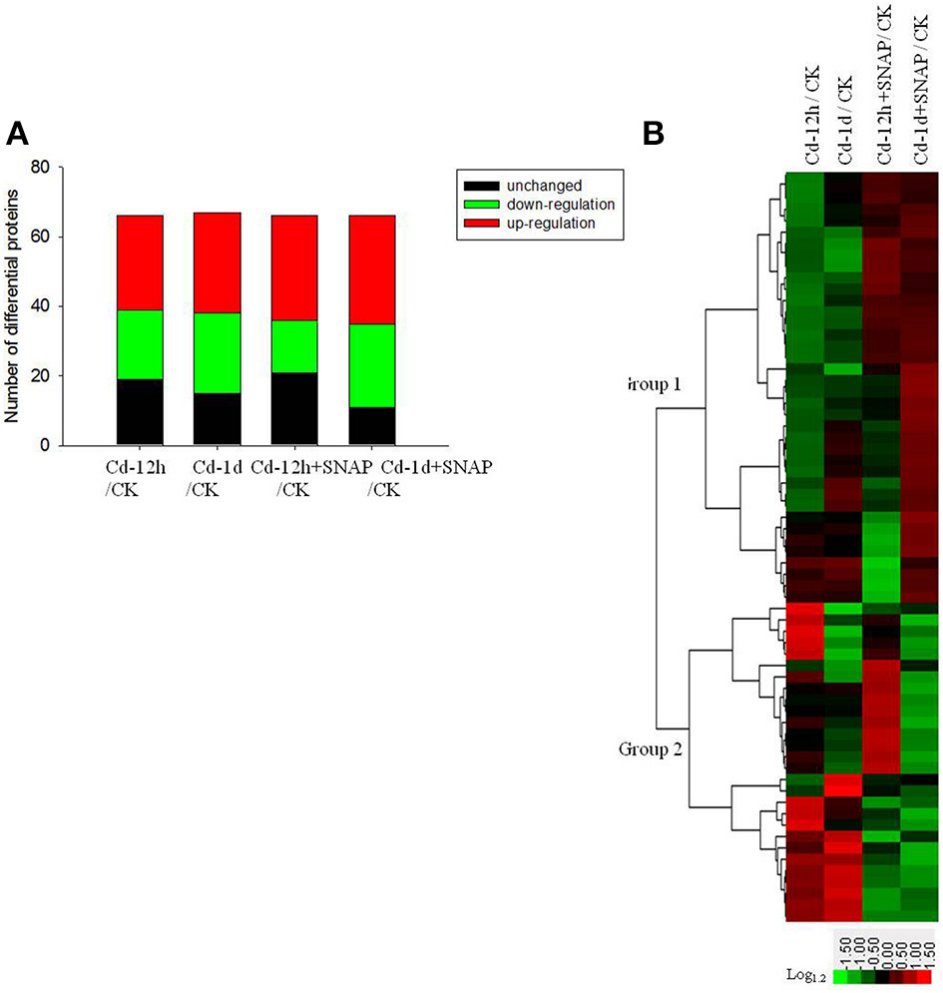
**Protein expression patterns in the total plasma membranes of rice seedling roots exposed to cadmium or cadmium and SNAP**. The total plasma membrane proteins from third-leaf-stage rice seedling roots were treated with 10 μM cadmium with or without 30 μM SNAP for 12 h and 1 day, and the differential expression protein were identified by the iTRAQ method. The number of up-regulated and down-regulated proteins were listed **(A)**, and hierarchical clustering of cadmium- and SNAP-responsive proteins were also presented **(B)**. The colors correspond to the log-transformed values of the protein change-fold ratios, as depicted in the bottom-right bar.

We determined the ratios of protein abundance for the following four groups: (1) Cd treatment for 12 h (12 h Cd)/the control line without treatment (CK), (2) Cd treatment for 1 day (1 d Cd)/the control line without treatment (CK), (3) Cd treatment for 12 h with SNAP (12 h Cd+SNAP)/the control line without treatment (CK), (4) Cd treatment for 1 day with SNAP (1d Cd+SNAP)/the control line without treatment (CK), to identify proteins affected by Cd or Cd and NO treatments. With a threshold of fold-change cutoff of 1.2-fold for increased accumulation and <0.6-fold for decreased accumulation, a total of 66 proteins showed differential accumulation (*p* < 0.05) when subjected to Cd or Cd and SNAP treatment as compared to the control line without treatment (Figure 4, Table 1, Supplemental Table 2). Among these differentially regulated proteins, 27 proteins were upregulated and 17 proteins were downregulated after 12 h of Cd treatment, 30 proteins were upregulated and 23 proteins were downregulated after 1 day of Cd treatment, 29 proteins were upregulated and 13 proteins were downregulated after 12 h of Cd and SNAP, and 31 proteins were upregulated and 24 proteins were downregulated after 1 day of Cd treatment and SNAP treatment. SNAP treatment did not increase the number of proteins showing up-regulation or down-regulation (Figure 4A), but rather only affected the expression intensities for these proteins. These identified proteins were divided into nine groups based on their biological functions (Table 1). The majority of these proteins were plasma membrane transporters (16), including nitrate transporters, phosphate transporters, oligopeptide transporters, a sucrose transporter, iron transporter, and a monosaccharide transporter etc. followed by ATPases (9) and kinases (9). Additionally, phosphatases and phospholipases (5), metabolism enzymes (6), antiporters (3), structural proteins (6), aquaporins (4), and signal and hormone related proteins (8) showed differential regulation. A hierarchical cluster analysis was conducted to categorize the proteins that showed differential expression profiles during Cd or Cd and SNAP treatment (Figure 4B). We found the expression profile for the identified proteins could be clustered into two groups. The proteins belonging to one group showed up-regulation after Cd treatment and the Cd+SNAP treatment could further enhance their upregulation. The proteins belong to the other group showed down-regulation and Cd+SNAP treatment further suppressed their accumulation. We also found most proteins that were aquaporins or involved in signal transduction and hormone response, were also differentially regulated by Cd or Cd+SNAP treatment (Table 1). Among these identified proteins, we observed one PLD protein that was up-regulated after Cd and Cd+SNAP treatments (Table 1-protein is highlighted in bold). Because it has not been reported before that PLD proteins could be localized to the plasma membrane after Cd or Cd and NO treatments, or that PLD protein accumulation could be regulated by Cd or Cd and NO treatments, these data hint that PLD has a function in the rice seedlings response to Cd stress. Thus, the role of PLD after Cd and Cd and NO treatments was further investigated.

**Table 1:** **List of proteins differentially regulated by Cd and Cd+SNAP in the plasma membrane as determined by using iTRAQ analysis**.

**Accession number^a^**	**Protein names**	**Cd (12 h): Control^b^**			**Cd (1 day): Control^c^**			**Cd**+**SNAP (12 h): Control^d^**			**Cd**+**SNAP (1 day): Control^e^**		
		**Rep 1 *p*-value Average** **Rep 2 *p*-value** **Rep 3 *p*-value**			**Rep 1 *p*-value Average** **Rep 2 *p*-value** **Rep 3 *p*-value**			**Rep 1 *p*-value Average** **Rep 2 *p*-value** **Rep 3 *p*-value**			**Rep 1 *p*-value Average** **Rep 2 *p*-value** **Rep 3 *p*-value**		
**ATPases**													
gi|218179	H-ATPase	1.664 1.586 1.624	0.037 0.023 0.034	1.625	1.769 1.770 1.742	0.013 0.005 0.006	1.760	1.926 1.800 1.871	0.038 0.035 0.037	1.866	1.932 1.961 1.926	0.004 0.005 0.009	1.940
gi|20302439	Plasma membrane H+-ATPase	1.292 1.269 1.397	0.017 0.009 0.008	1.319	1.384 1.541 1.440	0.021 0.032 0.023	1.455	1.493 1.477 1.420	0.033 0.003 0.021	1.463	1.689 1.699 1.667	0.046 0.005 0.008	1.685
gi|31432331	AAA family ATPase	0.811 0.843 0.838	0.041 0.012 0.032	0.531	0.971 0.887 0.859	0.003 0.036 0.008	0.706	0.986 0.940 0.994	0.034 0.009 0.012	1.273	1.510 1.457 1.684	0.003 0.004 0.008	1.550
gi|50252047	Putative calcium-transporting ATPase	0.850 0.785 0.794	0.006 0.009 0.008	0.510	0.956 0.805 0.883	0.016 0.009 0.021	0.881	0.850 0.983 0.955	0.029 0.028 0.001	0.929	1.611 1.633 1.603	0.042 0.035 0.021	1.616
gi|20302437	Plasma membrane H-ATPase	0.945 0.854 0.842	0.034 0.021 0.024	0.880	1.194 1.282 1.268	0.004 0.002 0.031	1.248	1.265 1.268 1.319	0.021 0.021 0.018	1.384	1.552 1.720 1.682	0.021 0.035 0.021	1.651
gi|544586339	Type IIB Ca^2+^ATPase	1.589 1.483 1.616	0.035 0.031 0.025	1.563	1.661 1.637 1.641	0.021 0.015 0.018	1.646	1.561 1.424 1.487	0.021 0.005 0.017	1.791	1.651 1.652 1.519	0.012 0.035 0.007	1.607
gi|77552962	Calcium-transporting ATPase 4	1.451 1.421 1.450	0.011 0.016 0.015	1.441	1.702 1.870 1.689	0.010 0.010 0.016	1.754	1.515 1.527 1.575	0.034 0.007 0.007	1.539	1.750 1.715 1.709	0.030 0.058 0.024	1.725
gi|31432100	Calcium-transporting ATPase 13	1.318 1.337 1.371	0.016 0.011	1.342	1.530 1.555 1.592	0.042 0.021	1.559	1.685 1.624 1.677	0.029 0.024	1.662	1.887 1.866 1.855	0.016 0.005	1.869
gi|122248711	ATP-citrate synthase alpha chain protein 2	0.554 0.552 0.637	0.030 0.022 0.025	0.581	0.445 0.453 0.442	0.021 0.027 0.021	0.447	0.554 0.552	0.005 0.020 0.023	0.369	0.554 0.552	0.006 0.025 0.011	0.369
**TRANSPORTERS**													
gi|23600439	Putative phosphate transporter OsPT1	0.777 0.592 0.624	0.017 0.020 0.021	0.664	0.573 0.498 0.480	0.043 0.035 0.017	0.517	0.854 0.687 0.583	0.027 0.028 0.025	0.708	0.829 0.736 0.835	0.009 0.005 0.007	0.800
gi|52550769	Phosphate transporter 7	0.762 0.778 0.744	0.011 0.020 0.007	0.861	0.798 0.759 0.771	0.048 0.021 0.035	0.576	0.678 0.646 0.648	0.036 0.036 0.032	0.657	0.474 0.509 0.482	0.010 0.021 0.009	0.488
gi|52550767	Phosphate transporter 5	0.514 0.492 0.454	0.003 0.028 0.025	0.487	0.434 0.442 0.345	0.017 0.021 0.021	0.407	0.505 0.511 0.499	0.006 0.007 0.008	0.505	0.480 0.489 0.545	0.038 0.035 0.032	0.505
gi|20279475	Putative ABC transporter	1.093 1.144 0.993	0.039 0.036 0.032	1.077	1.569 1.696 1.705	0.019 0.021 0.022	1.657	1.467 1.424 1.412	0.026 0.028 0.025	1.434	1.765 1.756 1.771	0.022 0.035 0.035	1.764
gi|37548736	Sucrose transporter SUT2	1.356 1.471 1.467	0.044 0.022 0.023	1.431	1.519 1.450 1.589	0.035 0.025 0.032	1.519	1.517 1.563 1.465	0.013 0.023 0.032	1.515	1.566 1.647 1.669	0.029 0.007 0.012	1.627
gi|49388481	PDR-like ABC transporter	0.439 0.461 0.433	0.030 0.014 0.010	0.444	0.669 0.543 0.460	0.023 0.016 0.023	0.557	0.660 0.622 0.757	0.021 0.011 0.007	0.680	0.724 0.699 0.790	0.001 0.002 0.010	0.738
gi|56785080	Putative oligopeptide transporter	0.841 0.843 0.866	0.021 0.028 0.024	0.850	0.642 0.771 0.753	0.025 0.025 0.010	0.722	0.495 0.482 0.513	0.028 0.002 0.010	0.497	0.400 0.503 0.446	0.006 0.013 0.011	0.450
gi|44953685	Iron transport protein 2	0.557 0.638 0.664	0.019 0.004 0.024	0.620	0.343 0.410 0.370	0.016 0.000 0.011	0.374	0.493 0.576 0.526	0.020 0.024 0.019	0.532	0.424 0.449 0.406	0.025 0.029 0.010	0.426
gi|108711111	Oligopeptide transporter 3	0.659 0.612 0.764	0.020 0.014 0.023	0.678	0.556 0.549 0.680	0.013 0.007 0.023	0.595	0.569 0.527 0.531	0.014 0.006 0.013	0.542	0.417 0.388 0.460	0.007 0.015 0.029	0.422
gi|3377509	Auxin transport protein REH1	0.413 0.499 0.371	0.006 0.013 0.009	0.428	0.627 0.624 0.721	0.009 0.002 0.014	0.657	0.449 0.468 0.496	0.026 0.012 0.027	0.471	0.352 0.462 0.333	0.023 0.003 0.001	0.382
gi|11991114	Monosaccharide transporter 3	1.494 1.584 1.445	0.012 0.017 0.021	1.508	1.563 1.660 1.666	0.009 0.002 0.027	1.630	1.534 1.524 1.545	0.009 0.000 0.027	1.534	1.764 1.711 1.771	0.016 0.018 0.016	1.749
gi|77549482	Nitrate transporter NTL1	1.143 1.220 1.342	0.007 0.023 0.018	1.235	1.454 1.656 1.564	0.026 0.013 0.016	1.558	1.443 1.420 1.454	0.029 0.019 0.017	1.439	1.643 1.620 1.656	0.007 0.033 0.005	1.640
gi|41052760	High affinity nitrate transporter	1.154 1.334 1.343	0.030 0.002 0.001	1.277	1.454 1.446 1.468	0.013 0.018 0.010	1.456	1.529 1.562 1,656	0.025 0.008 0.003	1.582	1.729 1.762 1.689	0.029 0.032 0.013	1.727
gi|6409176	Nitrate transporter	1.238 1.345 1.317	0.012 0.015 0.017	1.300	1.545 1.756 1.770	0.012 0.000 0.003	1.690	1.638 1.625 1.656	0.020 0.008 0.011	1.640	1.838 1.825 1.893	0.001 0.011 0.014	1.852
gi|41052964	Putative nitrate transporter NRT1	1.418 1.420 1.3032	0.011 0.015 0.027	1.380	1.623 1.725 1.740	0.032 0.028 0.014	1.696	1.585 1.520 1.567	0.002 0.033 0.024	1.557	1.785 1.720 1.756	0.014 0.021 0.029	1.754
gi|8547358	Phosphate transporter	0.649 0.626 0.643	0.030 0.021 0.029	0.639	0.798 0.759 0.771	0.048 0.021 0.035	0.776	0.549 0.526 0.534	0.030 0.019 0.006	0.536	0.454 0.504 0.454	0.006 0.011 0.025	0.471
**KINASES**													
gi|122247193	Benzothiadiazole-induced MAP kinase 1	1.319 1.214 1.324	0.031 0.021 0.020	1.286	1.565 1.634 1.650	0.014 0.023 0.022	1.616	1.519 1.514 1.565	0.018 0.005 0.025	1.533	1.619 1.614 1.656	0.007 0.006 0.020	1.630
gi|82654925	Calcium-dependent protein kinase isoform 11	0.554 0.552 0.554	0.018 0.010 0.003	0.318	0.454 0.452 0.483	0.029 0.024 0.009	0.463	0.654 0.652 0.656	0.029 0.015 0.009	0.654	0.676 0.656 0.670	0.018 0.006 0.019	0.518
gi|75148536	Probable calcium-binding protein CML7	1.319 1.214 1.342	0.010 0.015 0.004	1.292	1.445 1.434 1.446	0.012 0.010 0.029	1.442	1.519 1.514 1.545	0.003 0.021 0.009	1.526	1.619 1.614 1.703	0.012 0.013 0.017	1.645
gi|28564579	Putative receptor-like protein kinase	1.294 1.282 1.274	0.028 0.018 0.010	1.283	1.489 1.4998 1.4923	0.021 0.006 0.014	1.494	1.394 1.382 1.3656	0.015 0.021	1.381	1.594 1.526 1.567	0.015 0.009 0.031	1.562
gi|37534762	Putative serine/threonine-specific kinase like protein	0.547 0.547 0.524	0.005 0.029 0.002	0.539	0.667 0.637 0.635	0.018 0.021 0.020	0.646	0.547 0.547 0.523	0 013 0.023 0.031	0.539	0.434 0.443 0.452	0.028 0.023 0.031	0.443
gi|50725798	Putative protein serine/threonine kinase	0.757 0.768 0.756	0.013 0.024 0.003	0.560	0.747 0.748 0.893	0.026 0.007 0.029	0.796	0.847 0.848 0.867	0.022 0.012 0.005	0.654	0.412 0.484 0.448	0.005 0.016 0.020	0.448
gi|50938053	putative serine/threonine- specific protein	1.776 1.784 1.756	0.026 0.023 0.018	1.772	1.476 1.484 1.445	0.016 0.012 0.006	1.468	1.876 1.884 1.867	0.010 0.032 0.027	1.876	1.656 1.667 1.698	0.027 0.021 0.024	1.674
gi|34898856	Serine/threonine kinase receptor precursor-like	0.881 0.858 0.845	0.016 0.026 0.007	0.861	0.881 0.858 0.783	0.033 0.010 0.014	0.441	0.981 0.958 0.956	0.026 0.027 0.024	0.965	0.454 0.475 0.463	0.007 0.032 0.003	0.464
gi|38679443	Benzothiadiazole- induced somatic embryogenesis receptor kinase	1.234 1.256 1.223	0.003 0.010 0.022	1.238	1.454 1.434 1.440	0.033 0.010 0.014	1.443	1.434 1.456 1.461	0.023 0.018 0.007	1.450	1.634 1.656 1.715	0.016 0.009 0.016	1.668
**PHOSPHATASES AND PHOSLIPASES**													
gi|51090926	**Phospholipase D**	0.554 0.552 0.673	0.020 0.004 0.002	1.693	0.434 0.445 0.453	0.007 0.016 0.019	1.244	0.554 0.552	0.018 0.007 0.012	1.869	0.554 0.552	0.001 0.020 0.031	1.369
gi|34898886	Putative protein phosphatase 2C	0.554 0.552 0.454	0.016 0.018 0.022	1.220	0.654 0.652 0.656	0.021 0.030 0.032	1.654	0.554 0.552 0.545	0.014 0.027 0.013	1.550	0.454 0.445 0.436	0.031 0.024 0.035	1.645
gi|12698878	Phosphoinositide-specific phospholipase C	1.519 1.414 1.423	0.020 0.013 0.024	1.452	1.761 1.772 1.732	0.008 0.032 0.026	1.755	1.619 1.614 1.756	0.012 0.017 0.003	1.663	1.819 1.814 1.856	0.032 0.023 0.027	1.830
gi|223635541	Probable protein phosphatase 2C	1.519 1.214 1.323	0.014 0.010 0.014	1.352	1.656 1.656 1.670	0.032 0.005 0.021	1.661	1.519 1.514 1.546	0.025 0.024 0.005	1.526	1.619 1.614 1.676	0.005 0.009 0.021	1.636
gi|42408807	Putative phosphoprotein phosphatase PP7	0.554 0.552 0.565	0.023 0.020 0.022	0.857	0.554 0.552 0.487	0.008 0.024 0.007	0.631	0.554 0.552 0.576	0.011 0.017 0.027	0.461	0.444 0.443 0.434	0.028 0.017 0.016	0.340
**METABOLISM ENZYMES**													
gi|20374	Sucrose synthase	0.554 0.552 0.565	0.026 0.014 0.006	0.557	0.554 0.552 0.465	0.026 0.032 0.008	0.524	0.654 0.652 0.656	0.005 0.032 0.009	0.654	0.435 0.446 0.412	0.006 0.020 0.024	0.431
gi|22748323	Putative beta-1,3-glucanase	1.519 1.414 1.421	0.022 0.010 0.009	1.451	1.719 1.614 1.756	0.017 0.012 0.025	1.696	1.519 1.614 1.565	0.001 0.013 0.025	1.566	1.675 1.675 1.657	0.024 0.010 0.018	1.669
gi|34902210	Putative callose synthase	0.554 0.552 0.453	0.007 0.029 0.022	0.320	0.454 0.447 0.476	0.027 0.002 0.022	0.459	0.554 0.552 0.645	0.012 0.031 0.028	0.584	0.754 0.752 0.786	0.005 0.005 0.017	0.764
gi|50934423	Putative 3-glucanase	1.319 1.314 1.332	0.012 0.011 0.023	1.322	1.633 1.564 1.660	0.019 0.014 0.015	1.619	1.519 1.514 1.656	0.013 0.010 0.015	1.563	1.619 1.614 1.657	0.007 0.024 0.009	1.630
gi|50935131	Cellulose synthase-4	1.519 1.414 1.434	0.028 0.015 0.014	1.456	1.675 1.675 1.656	0.030 0.023 0.030	1.669	1.619 1.614 1.645	0.007 0.029 0.011	1.626	1.719 1.714 1.734	0.003 0.015 0.005	1.722
gi|4126809	Glyoxalase I	1.319 1.214 1.323	0.014 0.010 0.014	1.285	1.656 1.756 1.668	0.032 0.005 0.021	1.693	1.519 1.514 1.656	0.025 0.024 0.005	1.563	1.719 1.714 1.756	0.005 0.009 0.021	1.730
**Antiporters**													
gi|50251178	Putative Na^+^/H^+^ antiporter	0.680 0.780 0.705	0.013 0.026 0.021	0.722	0.492 0.451 0.422	0.033 0.021 0.022	0.455	0.824 0.903 0.843	0.009 0.030 0.004	0.857	0.932 0.924 0.978	0.010 0.027 0.031	0.945
gi|295048558	Sodium/proton antiporter	0.693 0.680 0.637	0.006 0.005 0.008	0.670	0.462 0.512 0.479	0.019 0.028 0.030	0.484	0.718 0.747 0.766	0.003 0.027 0.006	0.744	0.986 0.916 0.983	0.008 0.012 0.011	0.962
gi|57117296	Na+/H+ antiporter	0.814 0.714 0.703	0.001 0.021 0.015	0.744	0.413 0.495 0.464	0.004 0.022 0.001	0.457	0.734 0.853 0.825	0.011 0.032 0.030	0.804	0.908 0.895 0.915	0.005 0.005 0.023	0.906
**STRUCTURAL PROTEINS**													
gi|77548553	Auxin Efflux Carrier family protein	0.662 0.797 0.638	0.008 0.022 0.017	0.699	0.928 0.803 0.844	0.016 0.003 0.002	0.558	0.617 0.620 0.629	0.021 0.018 0.001	0.622	0.451 0.441 0.510	0.016 0.013 0.007	0.467
gi|541905597	Auxin efflux carrier 1b	0.676 0.645 0.749	0.033 0.024 0.028	0.690	0.520 0.461 0.438	0.016 0.003 0.022	0.473	0.701 0.720 0.763	0.023 0.017 0.010	0.728	0.818 0.822 0.823	0.008 0.021 0.007	0.521
gi|22831004	Plasma membrane intrinsic protein	0.718 0.726 0.784	0.009 0.019 0.004	0.543	0.818 0.826 0.867	0.021 0.001 0.006	0.837	0.618 0.626 0.656	0.023 0.010 0.025	0.633	0.445 0.454 0.446	0.022 0.009 0.014	0.448
gi|31193906	Putative plasma membrane intrinsic protein	0.719 0.702 0.645	0.029 0.018 0.026	0.689	0.454 0.504 0.446	0.001 0.019 0.023	0.468	0.719 0.702 0.767	0.020 0.030 0.029	0.729	0.919 0.902 0.945	0.028 0.018 0.017	0.922
gi|22831256	Putative plasma membrane integral protein	0.724 0.726 0.704	0.012 0.015 0.002	0.518	0.454 0.464 0.447	0.032 0.020 0.028	0.455	0.624 0.626 0.645	0.013 0.031 0.031	0.632	0.824 0.826 0.867	0.001 0.010 0.000	0.839
gi|385718822	ATP/ADP translocator protein	0.696 0.658 0.667	0.007 0.009 0.004	0.674	0.696 0.658 0.534	0.002 0.004 0.014	0.529	0.796 0.658 0.603	0.019 0.002 0.026	0.686	0.432 0.430 0.445	0.002 0.024 0.013	0.436
**AQUAPORINS**													
gi|3135543	Aquaporin	0.584 0.530 0.454	0.015 0.026 0.009	0.523	0.784 0.730 0.756	0.014 0.018 0.023	0.757	0.584 0.530 0.567	0.019 0.010 0.024	0.560	0.498 0.483 0.460	0.033 0.005 0.003	0.480
gi|108712233	Aquaporin PIP2.8	0.798 0.725 0.756	0.002 0.020 0.012	0.760	0.798 0.625 0.735	0.008 0.001 0.031	0.519	0.898 0.825 0.787	0.018 0.002 0.016	0.837	0.515 0.504 0.434	0.025 0.028 0.033	0.484
gi|110289400	Aquaporin NIP5.1	0.725 0.736 0.642	0.014 0.001 0.001	0.701	0.515 0.456 0.465	0.024 0.000 0.010	0.479	0.425 0.456 0.454	0.032 0.024 0.013	0.445	0.525 0.536 0.535	0.005 0.002 0.018	0.532
gi|110289278	Aquaporin PIP2.4	0.785 0.7120 0.723	0.026 0.017 0.031	0.540	0.885 0.820 0.856	0.017 0.024 0.020	0.854	0.685 0.620 0.745	0.013 0.010 0.010	0.583	0.464 0.454 0.465	0.020 0.029 0.021	0.661
**SIGNAL AND HORMONE RELATED PROTEINS**													
gi|52076930	Putative cyclic nucleotide gated channel homolog	1.474 1.468 1.498	0.003 0.029 0.012	1.480	1.674 1.668 1.656	0.007 0.021 0.033	1.666	1.474 1.468 1.465	0.016 0.012 0.026	1.469	1.412 1.434 1.356	0.010 0.031 0.015	1.401
gi|20190	Calmodulin	0.765 0.765 0.776	0.004 0.004 0.021	0.769	0.783 0.719 0.638	0.031 0.029 0.009	0.513	0.876 0.867 0.865	0.030 0.033 0.014	0.869	0.454 0.460 0.476	0.003 0.032 0.002	0.463
gi|7271253	14-3-3-like protein	0.454 0.452 0.472	0.026 0.005 0.015	0.459	0.765 0.778 0.745	0.005 0.014 0.027	0.763	0.554 0.552 0.545	0.014 0.032 0.009	0.550	0.754 0.752 0.756	0.024 0.027 0.023	0.754
gi|34897868	Putative extra-large G-protein	1.519 1.414 1.423	0.015 0.023 0.023	1.452	1.787 1.674 1.734	0.023 0.013 0.030	1.732	1.519 1.534 1.546	0.017 0.011 0.001	1.533	1.719 1.714 1.756	0.031 0.003 0.020	1.730
gi|13195452	GTP-binding protein	1.419 1.414 1.321	0.026 0.032 0.008	1.385	1.656 1.667 1.697	0.006 0.020 0.024	1.673	1.519 1.514 1.576	0.026 0.014 0.006	1.536	1.719 1.714 1.676	0.005 0.032 0.009	1.703
gi|6983881	Putative gibberellin response modulator	0.554 0.643 0.552	0.010 0.024 0.014	0.583	0.754 0.752 0.767	0.029 0.009 0.011	0.758	0.554 0.552 0.565	0.012 0.015 0.017	0.557	0.454 0.445 0.434	0.002 0.030 0.026	0.444
gi|75131369	Thioredoxin-like 4	0.554 0.552 0.483	0.005 0.007 0.013	0.530	1.504 1.502 1.553	0.027 0.003 0.017	1.520	0.854 0.852 0.845	0.030 0.024 0.025	0.850	0.348 0.346 0.334	0.016 0.022 0.024	0.343
gi|57899336	Thioredoxin family Trp26-like protein	0.519 0.714 0.645	0.012 0.000 0.024	0.526	0.389 0.378 0.3786	0.009 0.020 0.026	0.382	0.819 0.814 0.878	0.021 0.005 0.012	0.637	1.519 1.614 1.656	0.019 0.033 0.013	0.796

*Protein Phospholipase D was bold-marked for further analysis in this study*.

*The differentially regulated proteins induced by cadmium and cadmium and S-nitroso-N-acetylpenicillamine (SNAP) in enriched plasma membrane from rice analyzed after using isobaric tags for relative and absolute quantification (iTRAQ)*.

a *NCBI gene number ID*.

b *three repeated iTRAQ experiments between Cd treatment for 12 h and control without any treatment*.

c *three repeated iTRAQ experiments between Cd treatment for 1 day and control without any treatment*.

d *three repeated iTRAQ experiments between Cd treatment plus SNAP for 12 h and control without any treatment*.

e *three repeated iTRAQ experiments between Cd treatment plus SNAP for 1 day and control without any treatment*.

### Exogenous NO treatment promotes the translocation of PLD from the nucleus to the plasma membrane

**Figure 5:**
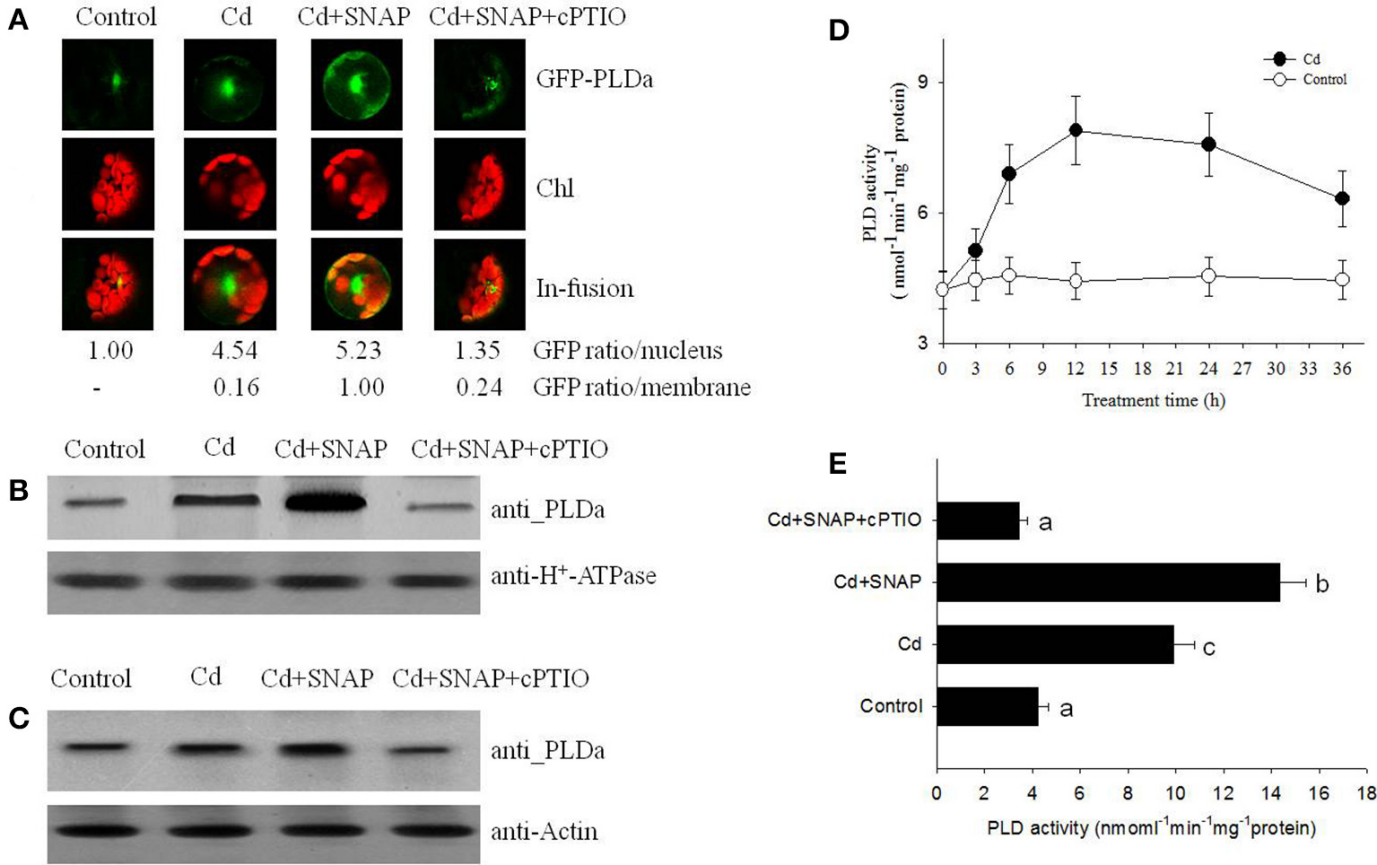
**NO promotes cadmium-induced PLD protein migration and its enzyme activity. (A)** GFP fluorescence in rice protoplasts transiently expressing PLDa-GFP fusion protein. Control, untreated; Cd, treated with 10 μM cadmium for 12 h; Cd+SNAP, treated with 10 μM cadmium plus 30 μM SNAP for 12 h; Cd+SNAP+cPTIO, treated with 10 μM cadmium plus 30 μM SNAP and 30 μM cPTIO for 12 h. From left to right: Flu, GFP fluorescence; BF:bright field; Merge (Flu+BF): merged image of FLU and BF. The GFP fluorescence intensity in the nucleus and membrane were quantified by Image J software (http://rsb.info.nih.gov/ij/download.html). **(B,C)** Western blotting and an anti-PLD antibody assay to detect PLDa accumulation in the enriched plasma membrane protein **(B)** and the total proteins **(C)**. Third-leaf-stage rice seedlings were treated with 10 μM cadmium with or without 30 μM SNAP or 30 μM cPTIO for 1 day. The enriched plasma membranes proteins and total proteins were isolated for PLD content analysis. **(D,E)** The rice seedling at three leaf stage were treated with 10 μM cadmium, the time course of PLD activity with (closed circle) or without Cd stress (open circle) were measured **(D)**, and the effect of different chemicals on PDL activity were measured after 1 day of treatment **(E)**. Values reflect means ± SEs of three independent experiments (*n* = 10/experiment). Different symbols above the bars indicate significant differences (Tukey's test, *p* < 0.05).

To understand the role of PLD during the rice response to Cd stress, we analyzed PLD localization in rice protoplasts transiently transformed with the vector expressing the pUBI:GFP-PLDa fusion gene. Prior to cadmium treatment, we observed strong GFP fluorescence in the nuclei before Cd treatment, indicating that GFP-PLDa was sequestered to the nuclei of transformed protoplasts before Cd treatment (Figure 5A). After 12 h of Cd treatment, a strong GFP fluorescence were also observed in the nuclei and around the plasma membrane, suggesting Cd treatment affected the expression of GFP-PLDa after Cd treatment (Figure 5A). We then found that addition of Cd and SNAP treatment further increased GFP fluorescence around the plasma membrane (Figure 5A). This effect was diminished by the NO scavenger cPTIO pretreatment. These data suggest that NO promotes the localization of PLDa to the plasma membrane, which is consistent with the proteomic data that Cd and Cd and SNAP treatment increased the accumulation of PLD in the plasma membrane (Table 1). Additionally, the anti-PLDa antibody was used to analyze PLDa protein accumulation in the total extracted protein or in the plasma membrane fraction from the seedlings after Cd and Cd+SNAP treatment. We found that Cd or Cd+SNAP treatments increased the level of PLD protein in the plasma membrane (Figure 5B) and partially increased the total PLD protein level in the total protein (Figure 5C). This increase in PLDa could be decreased in the PM and total protein fractions by pretreatment with cPTIO (Figures 5B,C).

We further measured PLD activity after Cd or Cd and SNAP treatment. Cd treatment induced PLD activity and PLD activity was highest between 12 and 24 h after Cd treatment (Figure 5D). Cd+SNAP treatment could further enhance the PLD activity, and the Cd+SNAP+cPTIO treatment (cPTIO is a NO metabolism scavenger) could suppress Cd or Cd+SNAP-induced increase in PLD activity (Figure 5E). These data correlated to our previous results and suggest that Cd and Cd+SNAP treatment could increase PLD protein levels and increase PLD enzyme activity.

### The cross talk of NO and PLD-mediated PA accumulation in rice seedlings under cadmium stress

**Figure 6:**
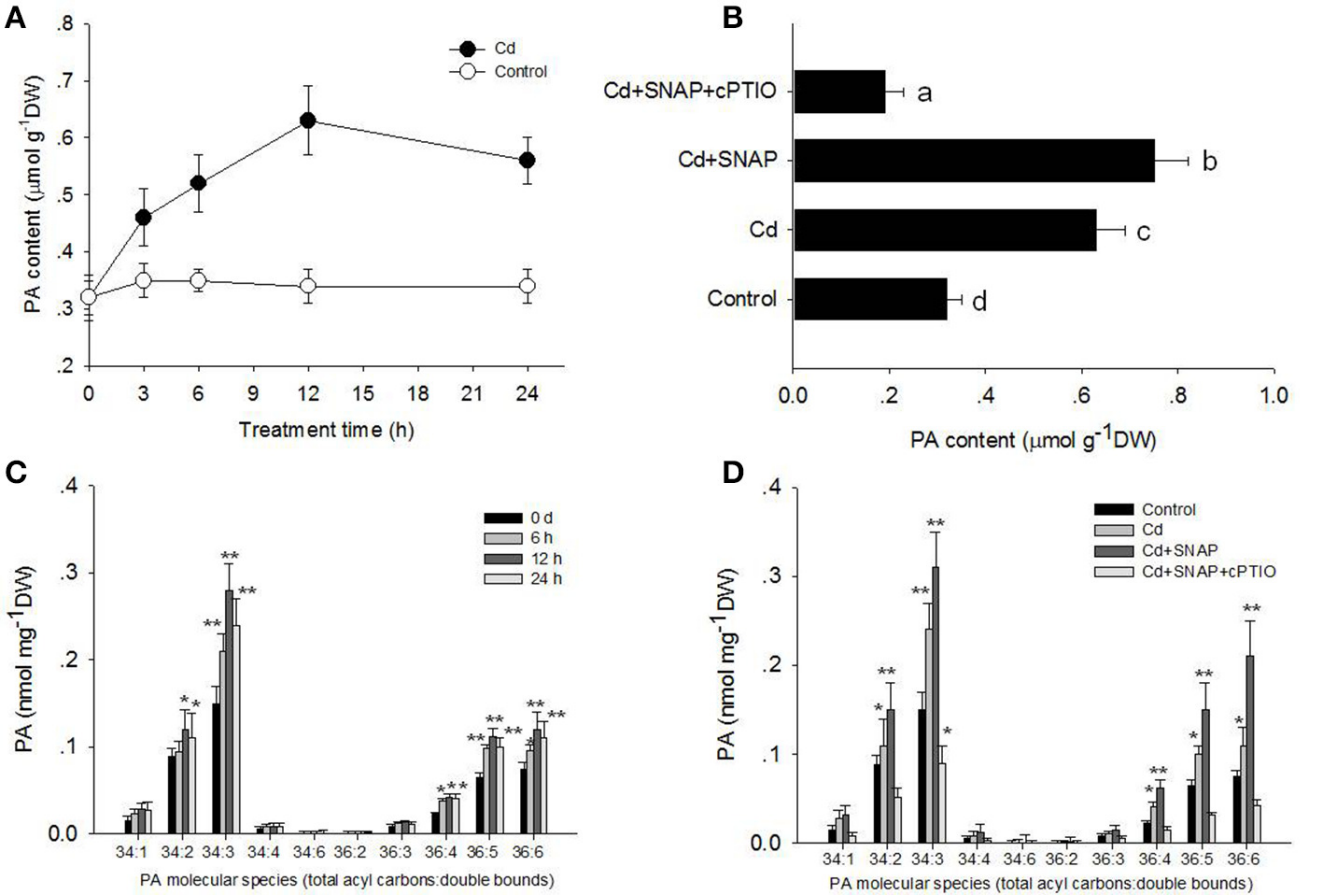
**Effects of cadmium, SNAP, and different chemicals on PA accumulation**. Third-leaf-stage rice seedlings were treated with 10 μM cadmium or other chemicals as above. The time course of total PA content with (closed circle) or without Cd stress (open circle) were measured **(A)**, and the effect of different chemicals on total PA content **(B)**. The different PA molecular species content was measured at the indicated times **(C)** and different PA molecular species content **(D)** were also measured after 12 h of treatment. The asterisk in **(C,D)** indicates that the mean value is significantly different from that of the control without any treatment (*P* < 0.05). Values reflect means ± SEs of six independent experiments (*n* = 10/experiment).

It has been previously shown that PLDa was activated in the ABA response and that phosphatidylcholine (PC) was hydrolyzed to PA in leaf protoplasts labeled with fluorescent PC (Zhang et al., 2004). Our above results showed that Cd and Cd and SNAP treatments increased PLD activity (Figures 5D,E). To further characterize the PA change in response to Cd or Cd and SNAP, we used electrospray ionization–tandem mass spectrometry (ESI-MS/MS) to analyze PA species in rice leaves. Cd induced a gradual increase in total PA, with a maximum increase occurring at 12 h after Cd treatment (Figure 6A). The addition of Cd and SNAP could further enhance total PA content but the effect could be suppressed by Cd+SNAP+cPTIO treatment (Figure 6B). Further analysis of PA molecular species revealed the distinguishable changes after Cd or Cd and SNAP treatment. The major molecular species of PA responsive to Cd treatment in rice seedlings were 34:2 (16:0-18:2), 34:3 (16:0-18:3), 36:4(18:2-18:2), 36:5 (18:2-18:3), and 36:6 (18:3-18:3; Figure 6C). The level of these PAs gradually increased after Cd treatment and reached the highest level after 12 h of treatment and decreased at 24 h after treatment (Figure 6C). Similarly, Cd and SNAP treatment could enhance, while Cd+SNAP+cPTIO treatment could suppress the Cd-induced increase of these PAs (Figure 6D). These results show that Cd and Cd and NO could induce 34:2, 34:3, 36:4, 36:5, and 36:6 PA accumulation through increased PLD activity.

### NO reduces cadmium-induced H_2_O_2_ generation and increases glutathione accumulation

**Figure 7:**
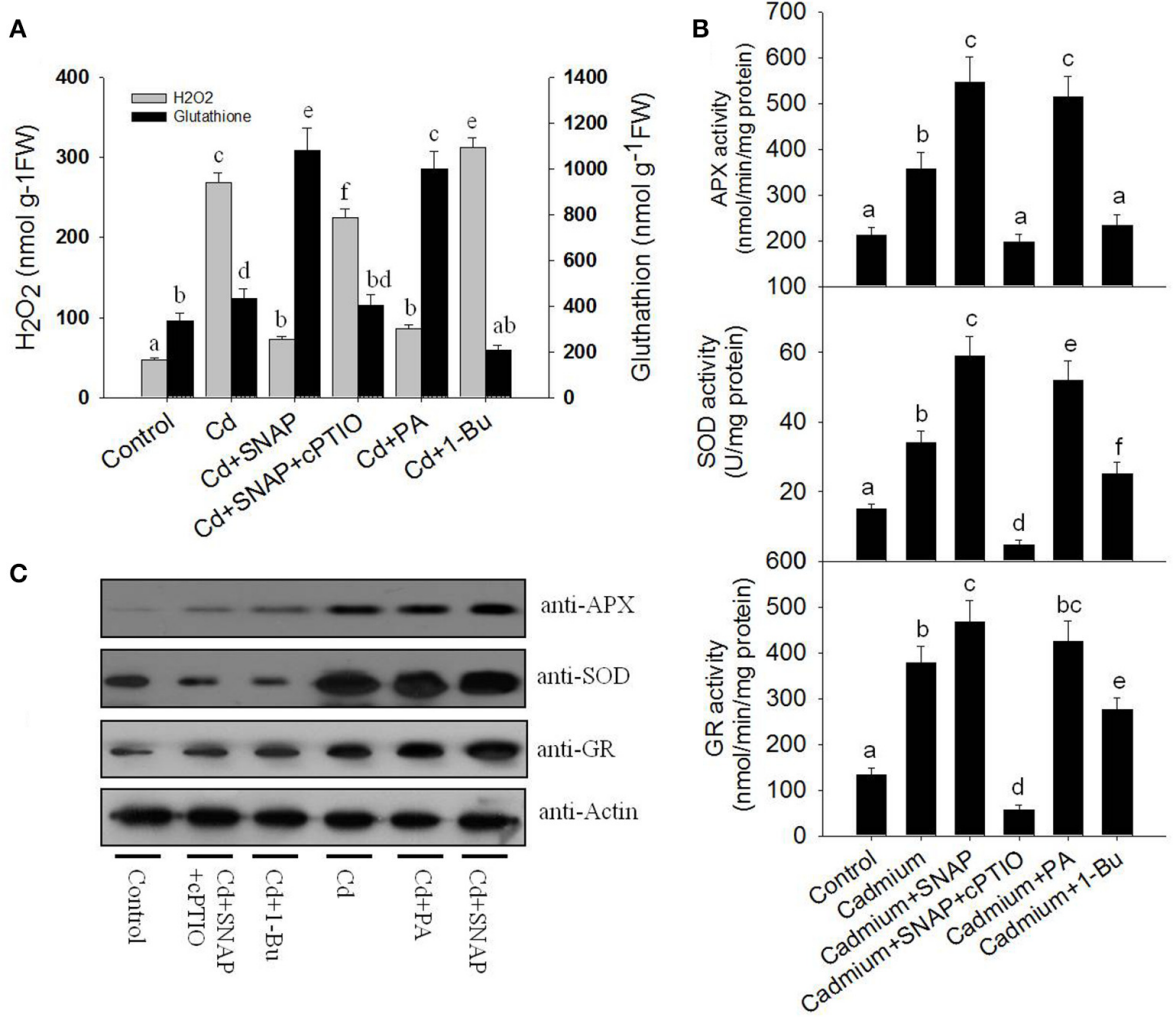
**NO and PA reduce cadmium-induced H_2_O_2_ accumulation and increase antioxidant accumulation. (A)** The effects of cadmium and different treatments on H_2_O_2_ and glutathione accumulation in rice seedlings. Rice was treated with cadmium, Cd and SNAP, Cd+SNAP+cPTIO, Cd+PA, and Cd+1-Bu. The content of H_2_O_2_ and glutathione were measured after 3 days of treatment. **(B)** The effects of cadmium, Cd and SNAP, Cd+SNAP+cPTIO, Cd+PA, and Cd+1-Bu on SOD, APX, and GR antioxidant enzymes activities. The asterisk in **(A,B)** indicates that the mean value is significantly different from that of the control without any treatment (*P* < 0.05). Values reflect means ± SEs of three independent experiments (*n* = 5/experiment). **(C)** The effects of cadmium, Cd and SNAP, Cd+SNAP+cPTIO, Cd+PA, and Cd+1-Bu on enzyme protein accumulations. The antioxidant enzyme activities and protein accumulation were measured after 3 days of treatments. Anti-actin antibodies were used as the loading control.

Heavy metal treatments can induce the production of large amounts of ROS, which damages cell viability (Shahid et al., 2014). NO treatment can reduce the damaging effects of ROS (Beligni and Lamattina, 2002). We then examined whether NO treatment could inhibit cadmium toxicity in rice seedlings by increasing antioxidant levels and reducing ROS damage. We measured H_2_O_2_ and glutathione levels in rice roots following cadmium or Cd and NO treatments. Cadmium exposure sharply increased H_2_O_2_ production (Figure 7A). The addition of Cd+SNAP reduced cadmium-induced H_2_O_2_ accumulation and sharply increased glutathione accumulation. This effect was abolished with Cd+SNAP+cPTIO pretreatment (Figure 7A). 1-Butanol (1-Bu) is the special inhibitor of PLD and suppressed PA production. We found that the addition of 1-Bu treatment increased the Cd-induced H_2_O_2_ accumulation and decreased glutathione accumulation. PA produced by PLD plays an essential role in many plant physiological process, such as stomatal closure, root growth, plant tolerance to salinity and water deficits, and nitrogen deficiency stress (Zhang et al., 2009). Here, we found that Cd and PA (mainly 16:0-18:2 PA) treatment also reduced Cd-induced H_2_O_2_ accumulation and enhanced glutathione accumulation. This suggests that NO and PA prevent Cd induced H_2_O_2_ accumulation and increase glutathione accumulation. The glutathione-ascorbate pathway is essential for scavenging ROS in plant cells (Foyer and Noctor, 2011). APX and GR are enzymes of the glutathione-ascorbate pathway. SOD also plays a role in reducing ROS over-accumulation. The addition of cadmium increased GR, SOD, and APX activities as compared to untreated plants (Figure 7B). We found that Cd and PA (16:0-18:2) and Cd and SNAP treatments further increased cadmium-induced activities of GR, SOD, and APX enzymes except for Cd and PA treatment for GR activity where the effect was not significant (Figure 7B). Additionally the Cd+SNAP+cPTIO and Cd and 1-Bu treatments reduced the cadmium-induced antioxidant enzyme activities (Figure 7B). Furthermore the enzyme levels were examined using antibodies to the APX, SOD, and GR enzymes (actin was used as a control; Figure 7C). As compared to the control plants, levels of APX, SOD, and GR increased with Cd, Cd+PA, and Cd+SNAP treatments. This increase in protein levels was abolished by the treatments Cd+SNAP+cPTIO and Cd and 1-Bu. Thus NO and PLD are required for the Cd-induced increase in these antioxidant enzymes.

We found that Cd treatment damaged rice seedling viability, including yellowish leaves, lower Fv/Fm and chlorophyll content, shorter shoot and root lengths, and higher ion leakage. However, the addition of NO or PA to cadmium treated rice seedlings could increase total chlorophyll content in leaves and reduce the negative effects on growth (Figures 1B,C). Suppressing PLD activity with 1-Bu, or NO (using SNAP) with cPTIO could increase Cd induced damage on cell viability (Figure 1A), including increasing ion leakage and further reducing Fv/Fm (Figure 1D), decreasing leaf chlorophyll content (Figure 1B), and decreasing the stem and root length (Figure 1C). These data suggest that both NO and PA play a role in relieving Cd damage to rice seedlings.

## Discussion

Cadmium is readily deposited into human and animal bodies after absorption and can damage the nervous system (López et al., 2006). In plants, cadmium reduces the absorption of nitrates and iron, damages photosynthetic capabilities, inhibits stomatal opening, and induces oxidative stress (Das et al., 1997; Lombi et al., 2002; Lin et al., 2007). Previous studies have primarily focused on physiological processes in the cytosol during cadmium stress in plants. The role of the plasma membrane in this process has received less attention, despite it being the initial site of cadmium sensing. We isolated and purified plasma membranes from 2-week-old rice seedlings and applied the quantitative iTRAQ method to investigate protein accumulation patterns following cadmium stress. Classification of the differentially accumulated proteins revealed these proteins were mainly membrane transporters, ATPases, and kinases and involved in catalytic function, suggesting the proteins involved in transport of ions and other molecules across membranes play an essential role during the response of rice seedlings to Cd stress.

Metal ions such as Cd disrupt the plasma membrane by binding to the proteins and lipids of the plasma membrane and can replace calcium ions in the membrane. This disruption to the plasma membrane increases non-specific membrane permeability and decreases specific transporting activities (Janicka-Russak et al., 2012). In this study, phosphate, iron, oligopeptide transporters in the plasma membrane were down-regulated whereas sucrose, monosaccharide, and nitrate transporters were up-regulated (Table 1). Ion homeostasis is regulated by the ATP dependent proton pump of the plasma membrane and Cd can reduce the enzyme activity of the H^+^ATPase (Janicka-Russak et al., 2012). H^+^ATPase, when in an active state, is bound to a 14-3-3 protein. In this study four H+ATPase proteins and a 14-3-3 protein in the plasma membrane were differentially regulated by Cd.

Exposure to Cd is known to increase the levels of jasmonate, abscisic acid, ethylene, auxin, and salicylic acid (Dalcorso et al., 2008; Chmielowska-Bąk et al., 2014). In this study an auxin transport protein REH1, two auxin efflux carrier proteins, and one gibberellin response modulator were all down-regulated after Cd treatment. This suggests that in rice seedlings that auxin or gibberellin may play a role in the Cd stress response in the plasma membrane.

In this study, six aquaporins in the plasma membrane were repressed by Cd stress. Water transportation through aquaporins might be involved in the tolerance and accumulation of Cd in pea (*Pisum sativum* L.; Belimov et al., 2015). The role of aquaporins in response to Cd may need further examination.

In response to Cd, ROS production can be triggered by calmodulin, protein kinases, phospholipase C, and phospholipase D (Chmielowska-Bąk et al., 2014). These phopholipases, through PA, activate secondary messengers such as lipid and protein kinases. Mitogen-activated protein kinases (MAPK) can affect transcription factors and thereby altering gene expression. In this study calmodulin, phospholipase C, phospholipase D, protein kinases including MAPK, and calcium dependent were differentially regulated in the plasma membrane. Based on the plasma membrane proteomic data, we propose that the rice cell initiated multiple strategies at the plasma membrane in response to Cd stress.

Previous studies showed that NO enhances the tolerance to cadmium stress in Arabidopsis and rice (Zhang et al., 2012; Yuan and Huang, 2016). PLD-dependent PA accumulation is also reported to alter plant response to salt, heat, and aluminum stress (Mishkind et al., 2009; Yu et al., 2010; Zhao et al., 2011). PLD activity is functional downstream of NO to control ABA-induced stomatal closure in Arabidopsis (Distéfano et al., 2012). Our proteomic data showed a PLD protein was upregulated substantially following cadmium treatment, and additional NO donor SNAP treatment could enhance PLD activity (Figure 5E). Western blotting confirmed that inhibiting NO generation reduced cadmium-induced accumulations of PLDa (Figure 5B), supporting the possibility of cross-talk between PLD and NO during cadmium exposure. Our physiological experiments have demonstrated that cadmium induces rapid generation of NO and the PLD product PA (Figures 3, 6). When NO accumulation was reduced by the NO scavenger cPTIO, cadmium-induced PA also was reduced (Figure 6). Applying rice with NO donor SNAP or PA reduced Cd toxicity to rice (Figures 1, 2), suggesting the critical roles of NO and PLD-mediated PA for rice tolerance to Cd stress. Interestingly, when PA was added to Cd treated seedlings, nitric oxide levels increased (Figure 3B) suggesting that the level of PA (via PLD) may affect the generation of NO. We also found that NO treatment promoted the translocation of GFP-PLDa from the nucleus to the plasma membrane. Such an effect could be abolished by NO scavenger cPTIO treatment. Furthermore, it is possible that NO promotes the localization of PLD to the plasma membrane, which is efficient strategy for the PLD product PA to easily bind to other important proteins. It is reported that PA may bind to NADPH oxidase to strictly controlling the ROS in Arabidopsis (Zhang et al., 2009). Thus PLD also plays a role in controlling ROS production in rice subjected to Cd stress.

ROS can induce cell death in Arabidopsis, of which, such effect could be reduced by adding PA (Zhang et al., 2003). Exogenous NO depletes Cd-induced toxicity by eliminating oxidative damage (Liu et al., 2015). Cd stress also caused the over accumulation of ROS in rice (Wang et al., 2015). Consistent with these reports, our data found that Cd treatment induced significant accumulation of H_2_O_2_, which damaged cell viability as shown by a reduction in Fv/Fm, biomass and phytochelatins. NO was rapidly induced by Cd treatment. NO also enhanced Cd-induced PLD activity and PA accumulation. Increases of NO or PA to cadmium-treated rice seedlings could increase antioxidant enzymes activities associated with glutathione-ascorbate pathway, including APX and GR activity, as well as the SOD activity, and reduced Cd-induced accumulation of ROS. Suppressing NO signal by a NO scavenger or PA generation by suppressing PLD activity increased the damage by Cd to rice seedlings. It also compromised the activities of APX, GR and SOD and their proteins accumulations. We also found increasing levels of NO or PA increased the content of glutathione (Figure 7). Glutathione was reported to contribute to control redox homeostasis under toxic metal and metalloid stress (Hernández et al., 2015). It is also the precursor of phytochelatins, which is induced by Cd stress in our study (Figure 2). These data coincide with previous studies and demonstrated that both NO and PA could reduce Cd stress by enhancing antioxidant protein accumulation and enzyme activities.

**Figure 8:**
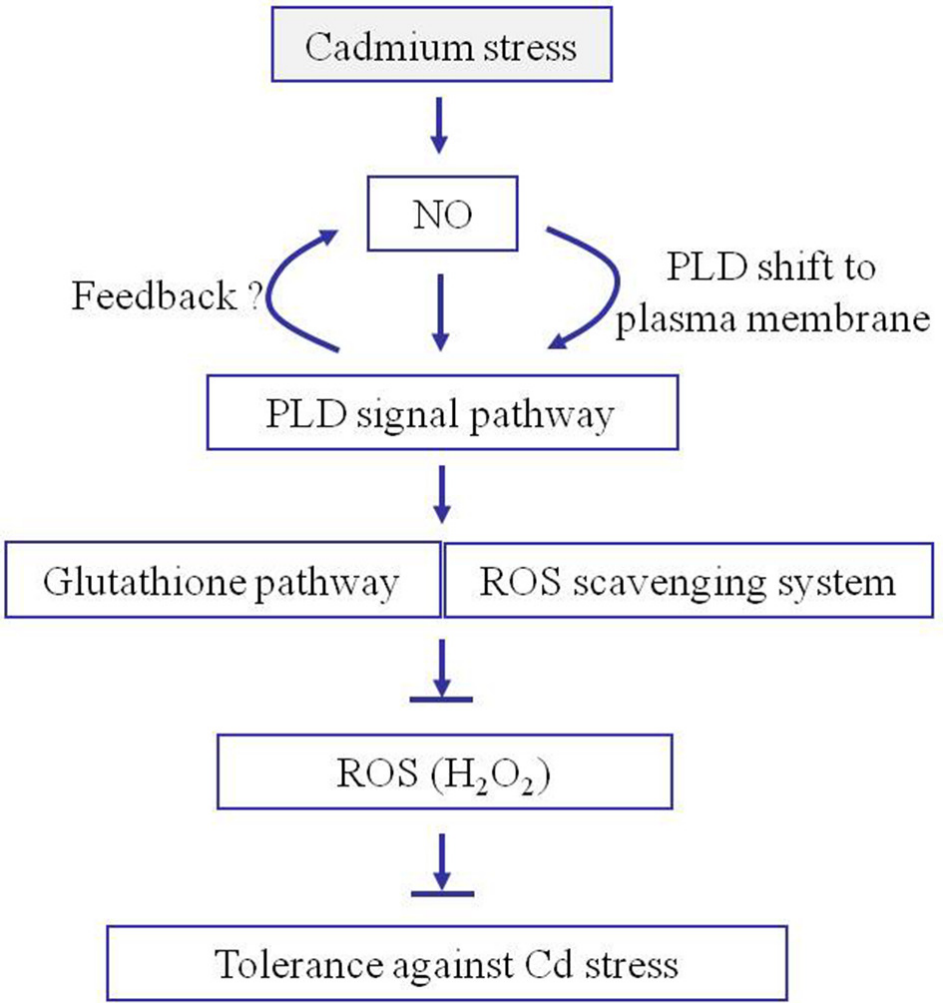
**Proposed model for the roles of NO and PA in facilitating rice seedling tolerance to cadmium stress**. Once subjected to Cd stress, NO is quickly generated in rice. NO increases the accumulation of PLD around the plasma membrane allowing PA to be generated. The PA induces the accumulation of antioxidants and reduces H_2_O_2_ accumulation. The accumulation of antioxidants and reduction in H_2_O_2_ increases the plant tolerance to cadmium stress.

Based on the proteomics and physiological results, we propose a model to explain the central roles of NO and PLD during the rice response to cadmium stress (Figure 8). Upon cadmium exposure, NO generation was rapidly induced to activate PLD activity and increase its accumulation, and accelerated its migration from the cell nucleus to the plasma membrane, which possibly lead to increased PA synthesis at the plasma membrane. Then PA acts as a signal to prevent the production of ROS and further induce the accumulation of antioxidant proteins and increase their activities. The accumulation of antioxidants and thus the reduction in ROS effectively protect against cadmium-induced toxicity. Altogether, our data support the multiple roles of the plasma membrane proteins during the rice response to Cd stress, and demonstrates the essential functions of NO and PLD-mediated signaling during this process. These findings should be useful to enhance rice tolerance to Cd stress through genetic modification of NO and PLD signaling.

## Conclusion

In this study, a quantitative proteomic approach was applied to obtain a comprehensive proteomic description of the plasma membrane from rice seedlings in response to cadmium stress or combining cadmium and NO treatment. Among the 66 differentially regulated proteins identified in the plasma membrane of rice seedlings, the majority of proteins were enzymes involved in metabolism, transporters, ATPases, kinases, phosphatases, and phospholipases. Damage symptoms induced by Cd stress, including morphological and biomass changes can be alleviated by NO or PA application. Addition of NO or PA resulted in the accumulation of glutathione and increased the activities of ROS scavenging enzymes, to alleviate cadmium-induced damage to rice plants. Addition of NO to Cd treated plants increased the synthesis of the Cd-responsive PA molecular species. Similarly, addition of PA to Cd treated plants increases nitrate oxide production. Taken together NO and PA serve a protective role in rice seedlings treated with Cd.

## Author contributions

LY and JJ performed the experiments, data analysis, and drafted the manuscript. KH, HW, HLW and EA assisted with data analysis, manuscript preparation, and revision. XH and YL served as the principal investigator, conceived the project, and finalized the manuscript.

### Conflict of interest statement

The authors declare that the research was conducted in the absence of any commercial or financial relationships that could be construed as a potential conflict of interest.

## Supplementary material

Supplementary Figure 1 **Effect of cadmium concentration on shoot and root growth of rice seedlings**.

Supplementary Figure 2 **Western blot measuring the enriched plasma membrane purity using different membrane marker antibodies**.

Supplementary Table 1 **The purity of the plasma membrane fraction was evaluated using marker enzymes associated with various subcellular membranes**.

Supplementary Table 2 **The detailed peptide information on proteins that are differentially regulated in the plasma membrane due to cadmium or cadmium and SNAP treatment**.
