# Supplementary figures and images for: The Dynamic Changes of the Plasma Membrane Proteins and the Protective Roles of Nitric Oxide in Rice Subjected to Heavy Metal Cadmium Stress
Source: Front Plant Sci. 2016 Feb 26;7:190. doi: 10.3389/fpls.2016.00190 (PMC4767926; doi:10.3389/fpls.2016.00190)

Supplemental Figure 1

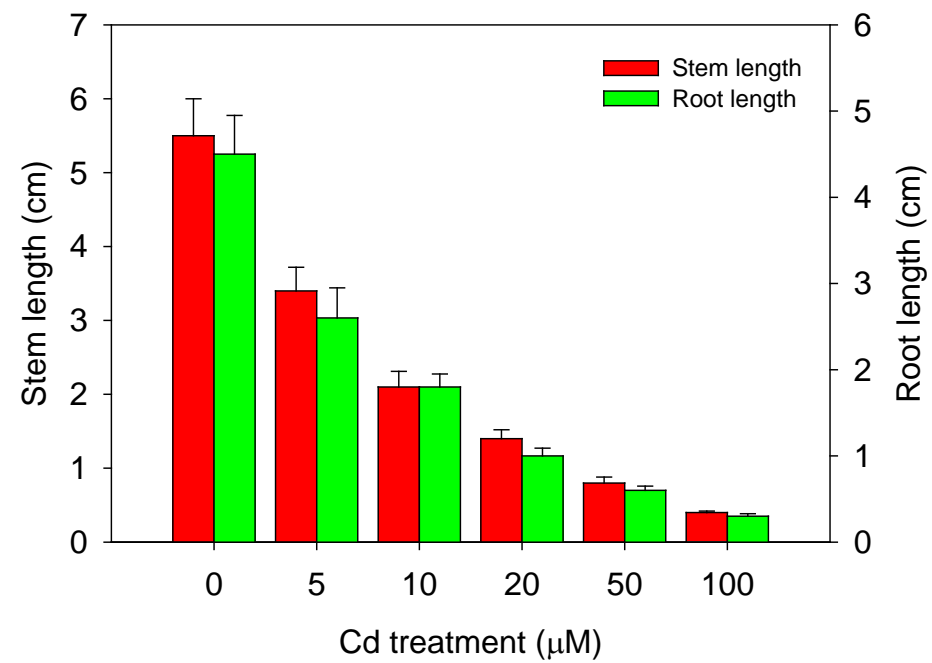

Supplemental Figure 2

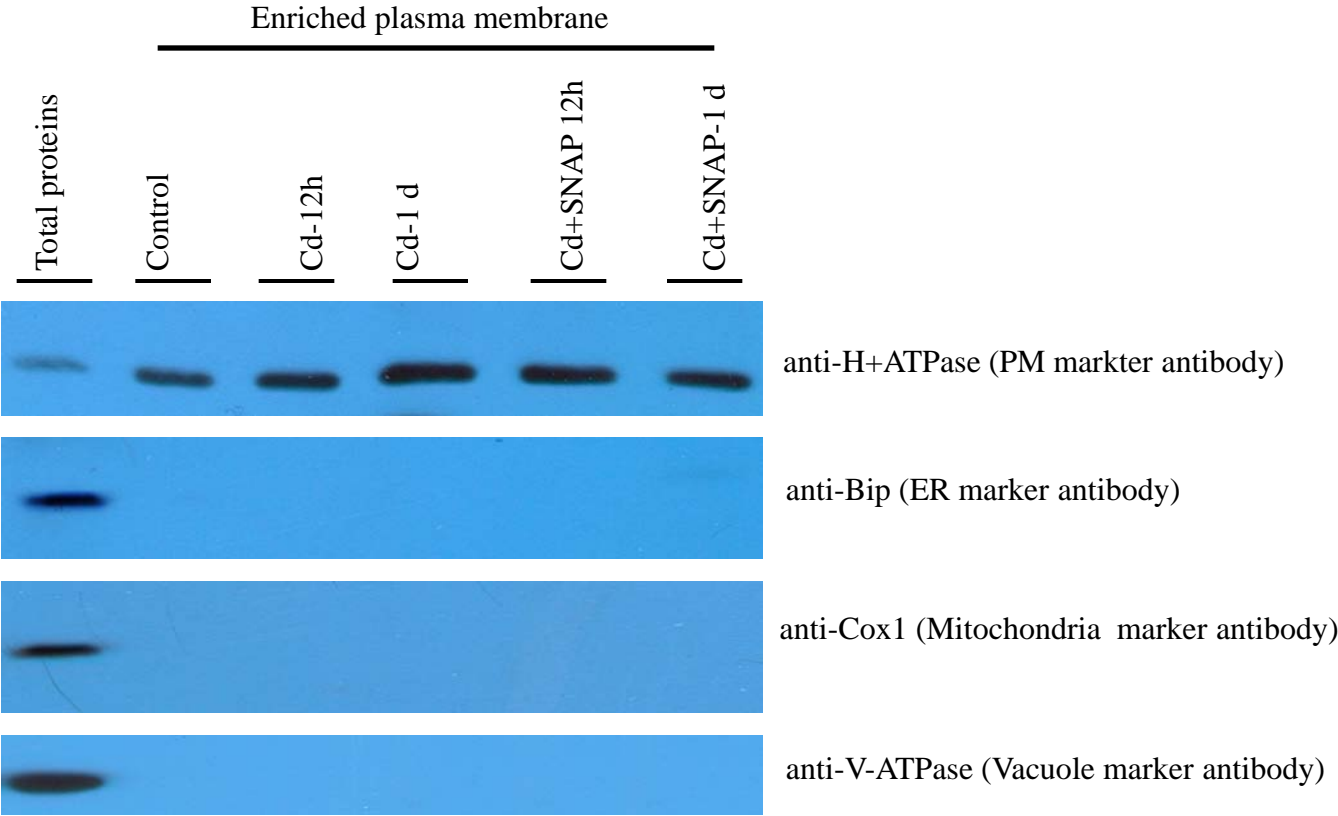

Supplement: Supplementary Figure 1 — Effect of cadmium concentration on shoot and root growth of rice seedlings. [file Presentation1.PDF]
