# Supplementary material for: The Dynamic Changes of the Plasma Membrane Proteins and the Protective Roles of Nitric Oxide in Rice Subjected to Heavy Metal Cadmium Stress
Source: Front Plant Sci. 2016 Feb 26;7:190. doi: 10.3389/fpls.2016.00190 (PMC4767926; doi:10.3389/fpls.2016.00190)
Supplement: Supplementary Table 1 — The purity of the plasma membrane fraction was evaluated using marker enzymes associated with various subcellular membranes. [file Table1.PDF]

Supplemental Table 1. The purity of the plasma membrane fraction was evaluated using marker enzymes associated with various subcellular membranes. Orthovanadate-sensitive ATPase, nitrate-sensitive ATPase, and azide-sensitive ATPase were selected as plasma membrane, vacuolar, and mitochondrial membrane markers, respectively. Three-leaf rice seedlings were treated with 30 μM cadmium with or without 50 μM SNAP for 3 days, and the plasma membranes were isolated from rice roots. The H^+-ATPase activity were measured with or without special inhibitors.

[a] The sensitivity of H+-ATPase activity to the enzyme inhibitors sodium orthovanadate, potassium nitrate and sodium azide was used to distinguish between plasma membrane, vacuolar membrane and mitochondrial membrane enzymes, respectively. Numbers shown are average (S.E. of 3 independent experiments.)

[b] The enzyme activity treated by indicated inhibitors.

**Control**			**10 μM Cadmium**			**10 μM Cadmium +30 μM SNAP**		
Treatment	H^+-ATPase[a]	Inhibited activity[b]	Treatment	H^+-ATPase[a]	Inhibited activity[b]	Treatment	H^+-ATPase[a]	Inhibited activity[b]
Control	2.76±0.14	-	Control	2.79±0.23	-	Control	2.48±0.15	-
Na_3VO_4	0.18±0.03	93.4%	Na_3VO_4	0.16±0.01	94.0%	Na_3VO_4	0.14±0.01	94.2%
KNO_3	2.59±0.12	5.8%	KNO_3	2.65±0.12	5.1%	KNO_3	2.34±0.12	5.6%
NaN_3	2.69±0.14	2.3%	NaN_3	2.69±0.11	3.5%	NaN_3	2.40±0.12	3.2%
